# Comprehensive Understanding of Closed Pores in Hard Carbon Anode for High-Energy Sodium-Ion Batteries

**DOI:** 10.1007/s40820-025-01833-x

**Published:** 2025-07-07

**Authors:** Siyang Gan, Yujie Huang, Ningyun Hong, Yinghao Zhang, Bo Xiong, Zhi Zheng, Zidong He, Shengrui Gao, Wentao Deng, Guoqiang Zou, Hongshuai Hou, Xiaobo Ji

**Affiliations:** College of Chemistry and Chemical Engineering, Central South University, Changsha, 410083 People’s Republic of China

**Keywords:** Hard carbon, Closed pores, Anode, Sodium-ion batteries, High energy density

## Abstract

Hard carbon (HC) is considered the most promising anode material for sodium-ion batteries (SIBs) due to its high cost-effectiveness and outstanding overall performance. However, the amorphous and intricate microstructure of HC poses significant challenges in elucidating the structure–performance relationship, which has led to persistent misinterpretations regarding the intrinsic characteristics of closed pores. An irrational construction methodology of closed pores inevitably results in diminished plateau capacity, which severely restricts the practical application of HC in high-energy-density scenarios. This review provides a systematic exposition of the conceptual framework and origination mechanisms of closed pores, offering critical insights into their structural characteristics and formation pathways. Subsequently, by correlating lattice parameters with defect configurations, the structure–performance relationships governing desolvation kinetics and sodium storage behavior are rigorously established. Furthermore, pioneering advancements in structural engineering are critically synthesized to establish fundamental design principles for the rational modulation of closed pores in HC. It is imperative to emphasize that adopting a molecular-level perspective, coupled with a synergistic kinetic/thermodynamic approach, is critical for understanding and controlling the transformation process from open pores to closed pores. These innovative perspectives are strategically designed to accelerate the commercialization of HC, thereby catalyzing the sustainable and high-efficiency development of SIBs.

## Highlights

- This review summarizes the latest advances in closed pore structures within hard carbon anodes for sodium-ion batteries, establishing a conceptual framework and origination mechanisms under a unified perspective of active sites.
- The influence of closed pore characteristics on sodium storage behavior is systematically explored, with design principles proposed for directional regulation of pore structures.
- Future research directions are highlighted, integrating advanced modification strategies with molecular-level design and dynamic/thermodynamic hybrid analyses for performance optimization.

## Graphical abstract

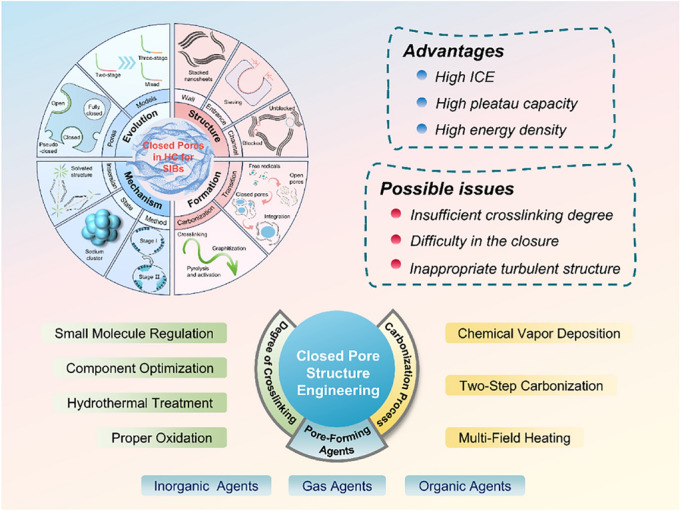

## Introduction

The development of efficient and cost-effective energy storage technologies is crucial for improving energy utilization and achieving sustainable development goals [1]. As global demand for renewable energy increases, the intermittency and instability of sources such as wind and solar energy present significant challenges. As a result, large-scale energy storage systems have become increasingly important, particularly battery energy storage systems [2–4]. Compared to lithium, sodium is much more abundant in the crustal content (Na: 2.36% vs. Li: 0.0065%) and has a lower production cost [5–9]. Therefore, sodium-ion batteries (SIBs) have become the preferred choice for meeting grid-scale energy storage requirements [10].

While SIBs offer advantages, including excellent low-temperature performance and high safety, challenges such as lower energy density, lower initial Coulombic efficiency (ICE), and poor rate capability continue to pose considerable barriers to further application [11]. Due to the large atomic radius of Na, there is a shortage of high-performance electrode materials for the anode, which remains a critical area in need of improvement and breakthroughs [12]. Among the reported anode materials for SIBs, organic-type materials are characterized by significant capacity decay, while conversion-type and alloy-type materials are hindered by substantial volume changes, both of which present clear limitations. Therefore, intercalation-type materials with stable cycling performance have garnered widespread attention. Among these, carbon-based materials are favored due to their higher theoretical capacities and greater abundance relative to titanium-based materials [13]. The sodium storage behavior across different carbon structures varies significantly. Na has limited intercalation ability in graphite, which features narrow spacing and an absence of defects. In contrast, Na easily intercalates into amorphous carbon, which possesses defects and expanded interlayer spacing, thereby forming stable compounds. Among these, hard carbon (HC) is considered to be the most promising and commercially viable anode materials, typically demonstrating a reversible sodium storage capacity of 300 mAh g^−1^ without modification [7, 14–16].

The complex charge–discharge profiles of HC can be divided into two distinct regions: the surface-controlled high-potential slope region (> 0.1 V) with excellent reaction kinetics and the diffusion-controlled low-potential plateau region (< 0.1 V) with relatively slower reaction kinetics [17]. The graphite layers, defects, and nanopores are considered key active sites in the microstructure, closely associated with electrochemical behavior of sodium storage. The slope region is widely regarded as crucial to enhancing power density. Defects, which deviate from the ideal graphene structure, are typically repaired at high temperatures, with the residual portion exhibiting pseudocapacitive adsorption behavior during electrochemical cycling [18]. However, excessively strong binding energies of some defects may irreversibly trap sodium ions and generate repulsive electric fields that interfere with other sodium storage behaviors. Additionally, the extensive surface area of the open pores leads to the formation of a large amount of solid electrolyte interphase (SEI), causing low ICE and reduced cycling stability [19, 20].

Due to the ambiguity between the adjacent carbon layer spacing and nanopores, the origin of the plateau region has remained a central topic of ongoing debate. Pseudo-graphite domains with interlayer distance (*d*_002_) in the range of 0.36–0.40 nm were once believed to be the primary source of plateau capacity [21–26]. Based on this, studies have also explored long-range ordered graphite regions (*d*_002_ < 0.36 nm) and highly disordered areas (*d*_002_ > 0.40 nm), highlighting how the effect of *d*_002_ on sodium storage behavior [26–29]. Although many studies have successfully controlled the *d*_002_ and defects, the active sites in the plateau region have not been significantly enhanced [29]. With the extensive expansion of precursors, closed pore structures that exhibit redox filling behaviors have been serendipitously identified. Closed pores with larger volumes can accommodate sodium atoms in high-density quasi-metallic sodium clusters, thereby maximizing their contribution to plateau capacity [30]. Consequently, the engineering of closed pores has become a major focus in the current research on HC modification, with the goal of constructing full cells that feature a wide voltage window and high energy density [31, 32]. In recent years, numerous high-quality strategies for the introducing and regulating of closed pores have been proposed, significantly improving overall performance and surpassing previous limits on reversible capacity. The prepared HC anode materials, with high ICE in the range of 80%–90%, are capable of providing reversible capacities ranging from 350 to 500 mAh g^−1^ [33–35].

Building on the development of various mechanisms, a variety of models have already been proposed [36]. The approach of identifying the mechanism within the plateau region based on characterization results has become a standard research procedure. However, it fails to provide a comprehensive analysis of the underlying causes of the observed discrepancies. Furthermore, the understanding of closed pore structures and storage mechanism remains unclear, particularly due to the absence of a rigorous scientific standard for distinguishing between open and closed pores. Simultaneously, the process through which open pores are generated and subsequently transformed into closed pores at high temperatures lacks a systematic explanation. These unresolved issues have resulted in the absence of a unified theoretical framework for various modification methods, significantly impeding the further development of SIBs with high ICE and superior energy density.

In light of these issues, this review commences by outlining the development history of pore structure and mechanisms, followed by an in-depth analysis of the significance of closed pore research. Subsequently, based on key pore structures such as curved nanosheets, pore entrances, and pore channels, the concepts of "pseudo-closed pores," "closed pores," and "fully closed pores" are introduced. These concepts help explain the differences between closed and open pores, as well as several phenomena observed during the closed pore filling process, including desolvation, steady state, and the underlying mechanisms. Finally, the entire process of pore structure formation is clearly described from the perspectives of molecular thermodynamics and kinetics. The review also provides a comprehensive summary of existing microstructural regulation methods from three key perspectives: cross-linking degree modulation, pore-forming agents, and carbonization process control, with the aim of offering systematic guidance for the advanced engineering of closed pores in HC materials. This review aims to assist researchers in comprehending the profound implications of the relationship between closed pore structure and electrochemical behavior in HC, thereby deepening their understanding of the overall microstructure. The detailed modification strategies outlined in this review serve as crucial references to advance the extensive research and practical application of HC in SIBs.

## Concept and Properties of Closed Pores

**Fig. 1:**
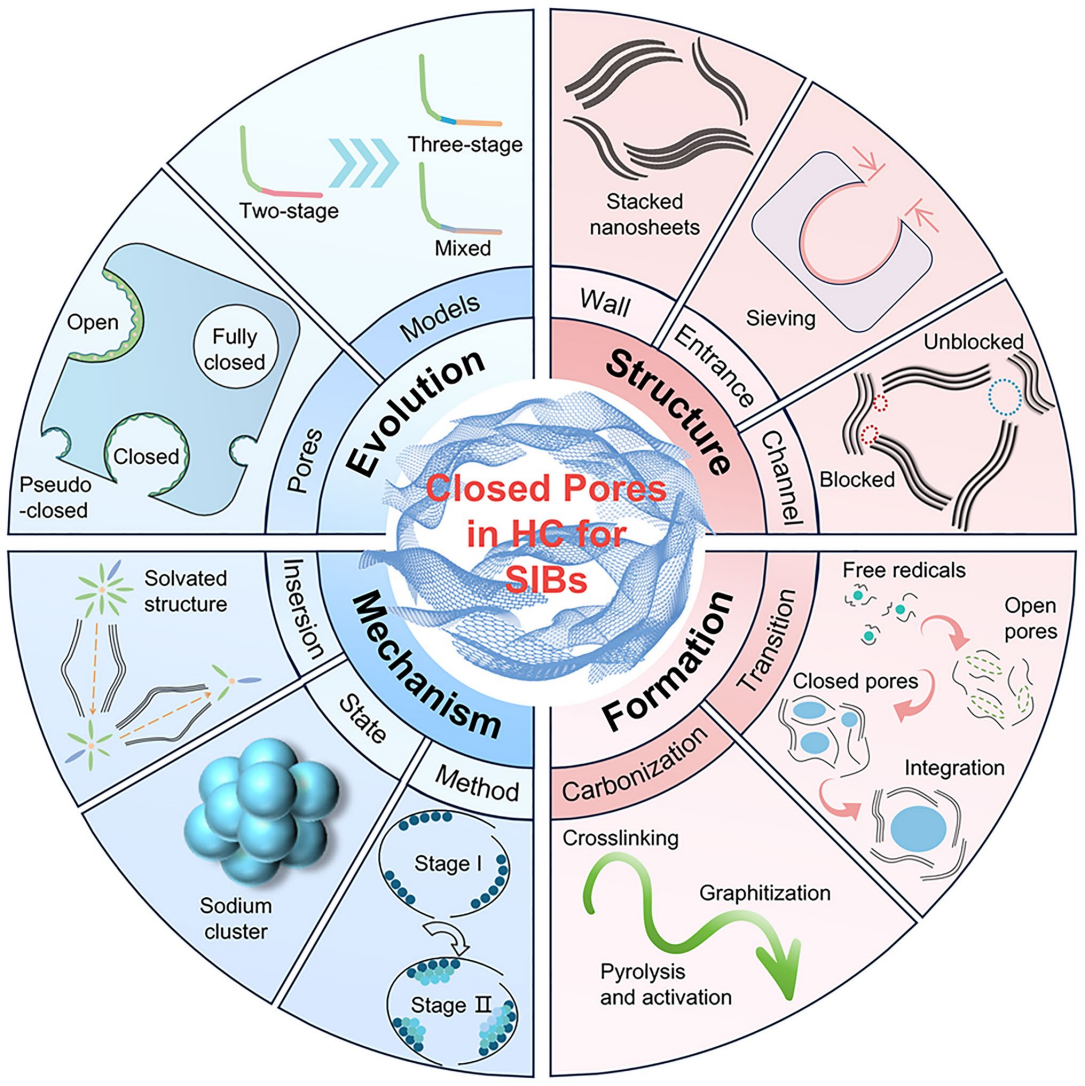
Schematic diagram of the evolution, structure, mechanism, and formation process of the closed pores in hard carbon of SIBs

Before designing and generating excellent closed pores, a comprehensive understanding of the concept and properties of closed pores is essential. Therefore, a detailed overview is provided to introduce the research history of pore structures, the definitions of different pore structures, the parameters of closed pores, the sodium storage mechanism, and the formation process (Fig. 1).

### Evolution of Nanopores and Associated Models

#### Two-stage Model Related to Pore Structure

**Fig. 2:**
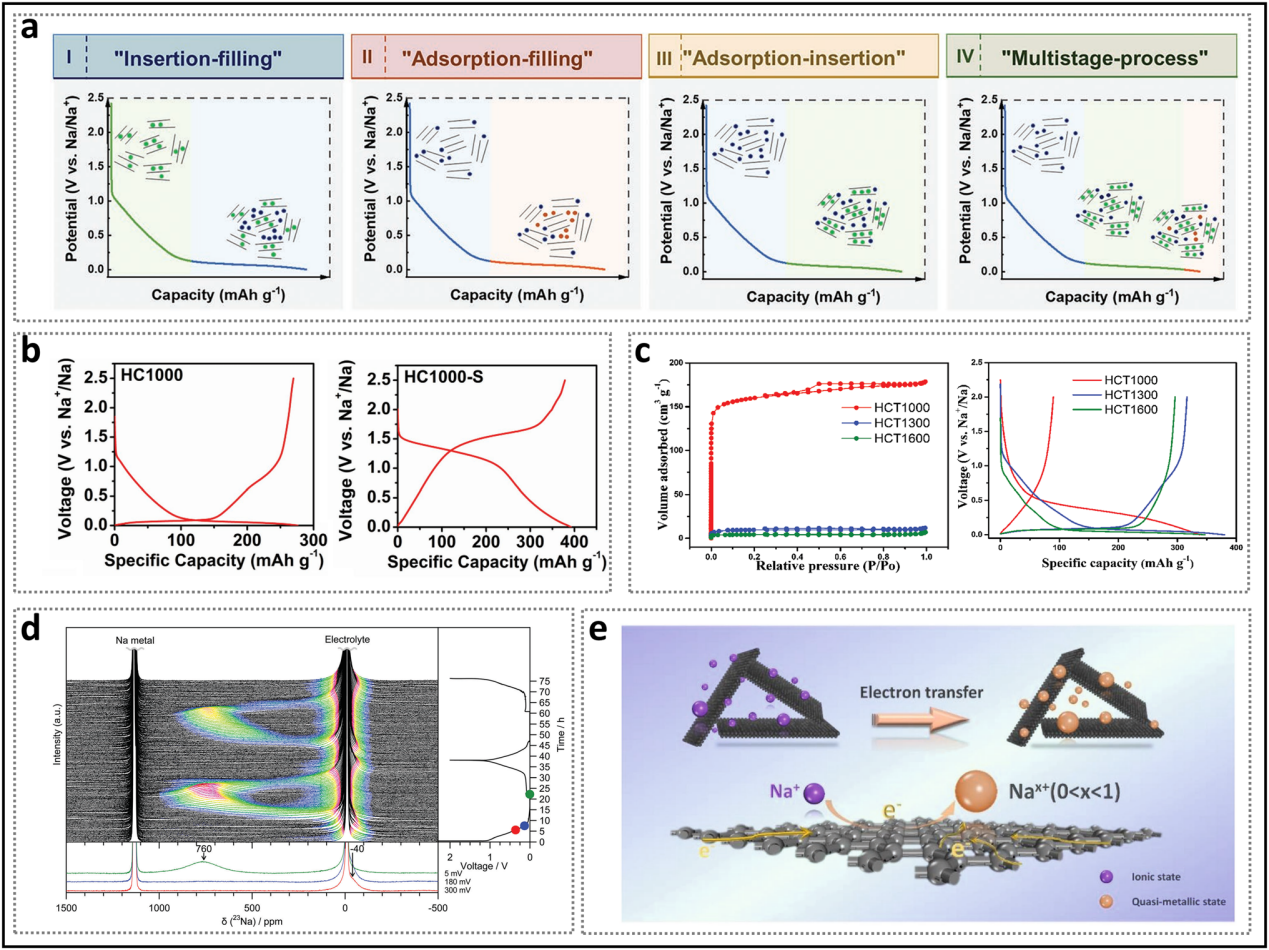
Evolution of mechanisms of sodium storage in closed pores. **a** Evolution of the two-stage models. Reproduced with permission [37]. Copyright 2022, Wiley. **b** Galvanostatic current charge–discharge curves before and after sulfur loading. Reproduced with permission [48]. Copyright 2018, Wiley. **c** N_2_ adsorption–desorption isotherms and charge–discharge curves of HC prepared at different temperatures. Reproduced with permission [49]. Copyright 2016, Wiley. **d** Operando ^23^Na NMR spectra for an electrochemical cell with sodium metal and hard carbon electrodes. Reproduced with permission [54]. Copyright 2016, The Royal Society of Chemistry. **e** Schematic illustration of the steady states of sodium in HC. Reproduced with permission [55]. Copyright 2021, Wiley

In recent years, the synthesis of HC with pore structures under various precursor and carbonization conditions has been intensively explored by numerous researchers, aiming to reveal the details of electrochemical curves and establish a clear process of the working mechanisms of HC in the sodium storage process (Fig. 2a) [37]. In 2000, Stevens and Dahn proposed the "intercalation–filling" mechanism (also known as the "intercalation–adsorption" model) [14]. This was based on their observations of the interaction between the pseudo-graphite layers and sodium ions in the slope region, revealed by wide-angle X-ray scattering (WAXS), as well as the changes in electronic density in the plateau region, observed via small-angle X-ray scattering (SAXS). This two-step creative approach suggested that the potential for sodium insertion into the nanopores is close to the deposition potential of metallic sodium. However, due to the limited characterization techniques available at that time, the behavior of sodium storage in the plateau region, whether involving adsorption or filling, was not fully elucidated [38]. The "House of Cards" structural model, which is based on these two types of active sites, has become widely accepted and has provided valuable insights into research on the structure–performance relationship [7, 39, 40]. The intercalation mechanism of Na in HC is proposed by analogy to that of Li in graphite, with the assumption that Na atoms require a larger *d*_002_ spacing [15]. For HC, defects are a significant factor responsible for the variation in the *d*_002_ spacing and facilitate the capture of sodium ion [41]. If defects are overlooked, the significant differences in carbon materials with respect to ICE, rate performance, and the ratio of slope to plateau capacity cannot be adequately explained, rendering the "intercalation–filling" model unconvincing [42]. Additionally, SAXS analysis of electronic density changes failed to effectively identify the specific mode of sodium atom storage in the pore structures, leading to ambiguity in the precise definition of the pores. In order to characterize the Brunauer–Emmett–Teller (BET) surface area and pore size distribution of the pore structure, N_2_ adsorption/desorption experiments have been widely adopted. As a result, researchers focused excessively on open pores, often categorizing them as supplementary defect adsorption sites. Well-developed pore structures were considered beneficial for improving ion diffusion rates but unfavorable for ICE. From this point onward, the “adsorption–intercalation” model, which applies to most HC materials, emerged as the mainstream view, while other pore structures that might influence the plateau region were largely overlooked [43]. What’s more, it is important to note that the steady-state intercalation of Na in HC (NaC_*x*_) remains unconfirmed, unlike Li in graphite (LiC_*x*_), and has even been contradicted [44–46]. Although several current observations suggest a potential intercalation mechanism, it remains a hypothesis that requires further in-depth exploration.

Since 2015, several experiments conducted at elevated carbonization temperatures have demonstrated a strong correlation between nanopore filling and plateau capacity [47]. Xu et al. [48] observed that doping sulfur to fill micropores resulted in the disappearance of the low-voltage plateau (Fig. 2b), thereby significantly enhancing the rationality of pore structure filling. The emergence of the “adsorption–filling” model naturally attracted considerable attention from researchers, as it contrasts with the conventional understanding of the role of open pores play in influencing electrochemical performance. Furthermore, carbonization temperature, a critical parameter that induces significant changes in pore structure properties, has been extensively discussed. Titirici et al. [49] found that, during the heating process from low to high temperatures, the calculated BET surface area dramatically decreased (Fig. 2c), and the reversible capacity transitioned from being dominated by the slope region to being dominated by the plateau region. The introduction of true density measurements further supported this view. Hasegawa et al. [50], by calculating the carbon skeleton density and pore volume of samples with small BET surface areas at temperatures above 1600 °C, found that they still contained a significant number of closed pores, which contributed to plateau capacity. Hu et al. [51], employing various pore structure characterization techniques, demonstrated that plateau capacity was positively correlated with both the pore diameter and volume of closed pores in a series of materials derived from naturally porous softwoods. Since then, open and closed pores, exhibiting distinct characteristics in the microstructure of HC, have been discussed separately. Simultaneously, the understanding of stable state of sodium in closed nanopores has gradually evolved. Due to the low potential of the plateau region, some views suggest that the strong metallic state in closed pores may lead to an increased risk of sodium plating in HC, while others counter this argument, asserting that sodium does not exist in the form of metallic sodium, as no sodium plating phenomenon has been observed [47]. Moreover, some studies extended the use of ^23^Na magic-angle spinning (MAS) solid-state NMR (ssNMR) technology to characterize HC anode materials for SIBs, which revealed that sodium in the pore structures exists entirely in ionic form [52, 53]. In 2016, Stratford et al. [54] were the first to observe the reversible appearance of peaks with partial metallic characteristics in the plateau region using *operando* ^23^Na ssNMR and ex situ ^23^Na MAS ssNMR spectra (Fig. 2d). This strongly suggests the existence of closed pores containing steady-state quasi-metallic sodium (Na^*δ*^, *δ* < 1) (Fig. 2e) [55]. Subsequently, additional relevant studies have supported this viewpoint, indicating that in larger pore structures, quasi-metallic sodium clusters form, which play a decisive role in prolonging the low-voltage plateau [56, 57].

#### Open Pores and Closed Pores

From a size perspective, nanopores encompass small mesopores (> 2 nm), micropores (< 2 nm), and ultramicropores (< 0.7 nm). In addition to pore size, pore structures are further classified into open and closed pores based on the degree of pore openness. Open pores, regardless of their location near the surface or deep within the material, feature large pore entrances and channels that are directly connected to the external environment. This characteristic renders them highly accessible to various gases and liquids, thereby facilitating adsorption and chemical reactions. Consequently, simple adsorption/desorption experiments are extensively employed in research to derive adsorption/desorption isotherms. From these curves, the specific surface area (*S*_BET_) can be determined using the BET method, which depends on the pore geometry and size. N_2_ is widely applicable due to its cost-effectiveness and ability to provide pore information larger than 1 nm. These open pores typically exhibit type I or type II adsorption/desorption isotherms, corresponding to micropores and mesopores, respectively. In some HC, a mixture of both isotherm types can be observed, suggesting the presence of heterogeneous pore sizes resulting from the combination of micropores and mesopores [51, 58]. Occasionally, a noticeable hysteresis loop is observed in the mesopore isotherms, which is primarily attributed to capillary condensation, a process that directly leads to gas–liquid phase transitions [59–61]. Additionally, CO_2_, with a smaller molecular diameter (3.3 Å for CO_2_ vs. 3.64 Å for N_2_), can be employed to effectively detect smaller micropores and ultramicropores (< 0.7 nm) at higher working temperatures (CO_2_ at 273 K vs. N_2_ at 77 K) [62]. By combining these two gases, the overall pore size distribution of porous materials can be accurately determined, providing valuable guidance for pore design.

During the sodium insertion process, sodium ions are gradually diffused through the electrolyte. When they reach the negative surface of the HC material, they adsorb and form an interfacial electric double layer, creating a favorable environment for subsequent electrochemical reactions. As the potential decreases to a lower range, electrons are transferred from the electrode to the lowest unoccupied molecular orbital (LUMO) of the electrolyte molecules, causing an irreversible reductive decomposition of the electrolyte. As the potential continues to decrease, a SEI forms on the electrode surface, preventing further decomposition of the electrolyte. These two processes correspond to two irreversible reduction peaks in the cyclic voltammetry (CV) curve during the first cycle [63]. In characterization, the SEI on the particle surface can usually be observed through high-resolution transmission electron microscopy (HRTEM), appearing as regions that are distinct from the interior of the carbon material. Furthermore, compared to ester-based electrolytes, which may form SEI with thicknesses of tens of nanometers, ether-based electrolytes form dense and thin SEI (< 10 nm), leading to superior interface stability and rate performance [64]. In addition to the external surface of the particles, the significant porosity within the particles often results in a high *S*_BET_ [65]. This implies that the electrolyte will inevitably enter the open pores, leading to a very low reversible capacity and ICE. Although researchers are aware that the specific surface area inside the material is equally important, traces of the inner surface SEI are difficult to distinguish. Consequently, the effects of the inner surface SEI are often analyzed together with those of the outer surface. Therefore, despite the potential of multi-stage pore structures with a broad distribution to accelerate ion transport dynamics and enhance rate performance, there is increasing recognition that only constructing open pores is not a satisfactory approach [66]. The introduction of the emerging closed pores, which is formed at high temperatures, has made it possible to achieve low *S*_BET_ and high ICE for HC. At the same time, the closed pores strategy imposes stricter requirements on structural issues and surface physicochemical analyses.

Due to the limitations inherent in the HRTEM projection imaging mode, the highly random, turbulent structure of three-dimensional carbon materials is largely obscured, which complicates the distinction between open and closed pores [49]. Historically, research has primarily focused on the surface properties of pore structures, assuming that closed pores are forms of pores that cannot be detected by gases such as N_2_ and CO_2_. Based on this view, two-dimensional diagrams from some researchers depict closed pore structures as fully enclosed, formed by interconnected microcrystalline walls with no dead ends. However, this does not accurately represent the true nature of closed pores. Some studies suggest that HC with engineered closed pores exhibits higher ICE and plateau capacity, even when the *S*_BET_ measured by N_2_ is large or exceeds that of similar materials [59, 67, 68]. Some studies suggest that these pores may contribute to enhancing plateau capacity, thus challenging conventional perceptions regarding their role [69]. These findings suggest that the characteristics of closed pores warrant further investigation. While adsorption/desorption experiments can serve as an initial criterion for identifying closed pores, they are not always rigorous in certain aspects, which limits the advancement of pore structure construction strategies based on theoretical insights. In contrast, categorizing open and closed pores based on their ability to provide reversible plateau capacity represents a more effective approach, as HC is in direct contact with and operate in the electrolyte. More specific information on closed pores will be discussed in the next section.

#### Continued Supplementation of the Three-Stage Model

**Fig. 3:**
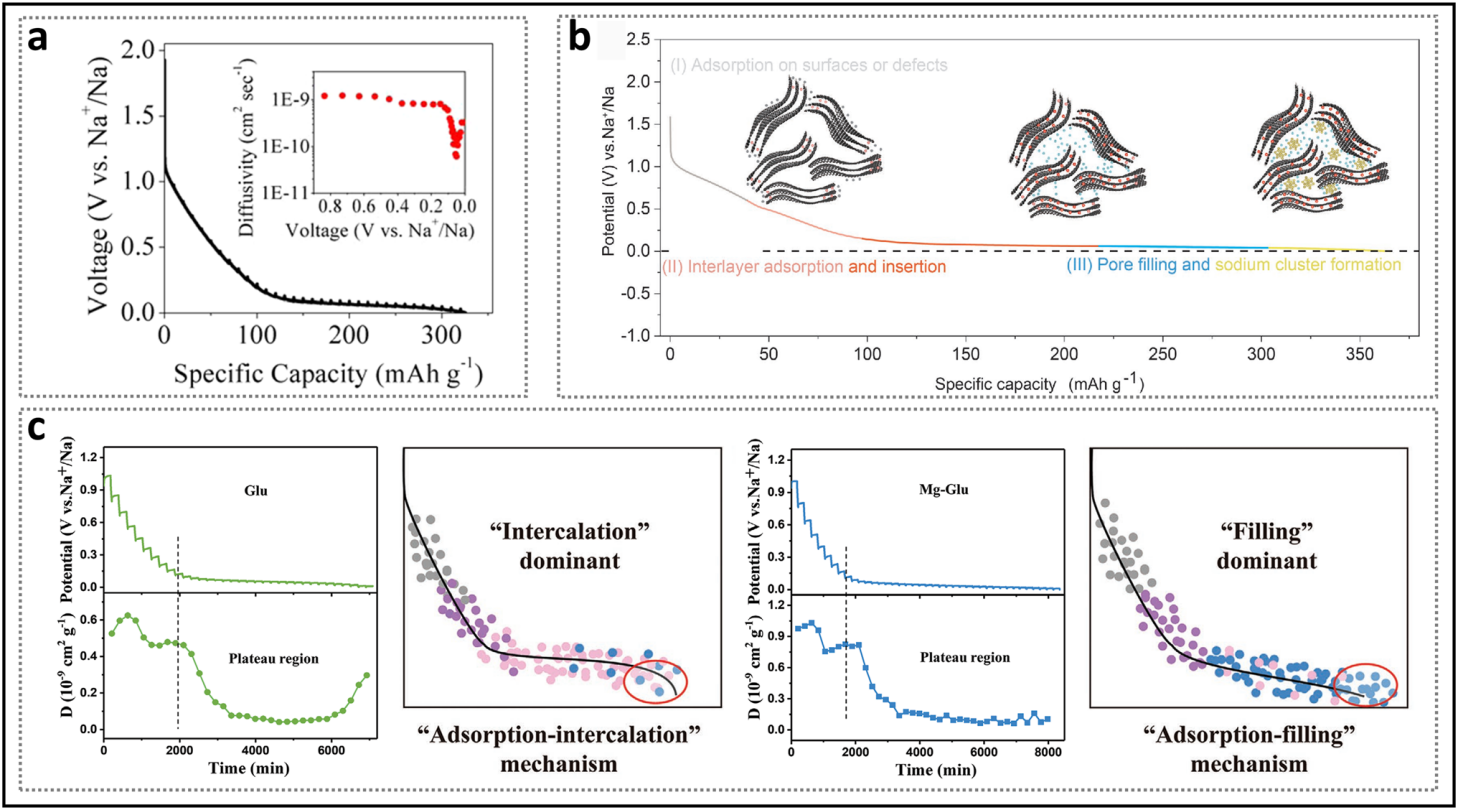
Introduction of the three-stage model. **a** GITT profile and diffusivity as a function of states of charge. Reproduced with permission [70]. Copyright 2015, American Chemical Society. **b** Schematic diagram of the sodium storage stages of HC. Reproduced with permission [60]. Copyright 2023, Wiley. **c** Different GITT profiles and their apparent diffusion coefficients, along with the corresponding dominant mechanisms. Reproduced with permission [59]. Copyright 2022, Wiley

Alongside the “adsorption–filling” model, the composite model has also attracted significant attention. In 2015, Bommier et al. [70] introduced a three-stage “adsorption–intercalation–filling” model. They observed that the sodium-ion diffusion coefficient of the prepared HC exhibited three distinct stages as the discharge depth increased (Fig. 3a), utilizing the galvanostatic intermittent titration technique (GITT): (i) The slope region maintained a diffusion coefficient of approximately 10^−9^ cm^2^ s^−1^, corresponding to sodium-ion adsorption at defects, (ii) in the plateau region, the coefficient drastically decreased by about one order of magnitude and then gradually leveled off, and (iii) in the latter half of the plateau region (from 0.05 V to cutoff voltage), it unexpectedly increased. This phenomenon led some researchers to speculate that HC materials simultaneously satisfy the requirements of both intercalation and filling mechanisms. Theoretical calculations indicated that once sodium ions accumulate densely in the plateau region, repulsive forces increase, resulting in a decrease in diffusion coefficients between 0.1 and 0.05 V [40]. Some studies have suggested that sodium cluster formation initiates once the diffusion coefficient reaches an inflection point [18, 71]. Building on support for the three-stage theory, Chou et al. [60] proposed the "adsorption–intercalation–pore filling–sodium cluster formation" model as an extension (Fig. 3b). As the discharge depth increases, sodium ions first fill the pore walls away from the core and then aggregate into clusters at the center. During the formation of sodium clusters, sodium ions are progressively reduced, leading to a decrease in interionic repulsion, thereby providing a plausible explanation for the observed inflection in diffusion coefficients. For HC materials, whether sodium intercalate as NaC_*x*_ or form quasi-metallic sodium within nanopores, they are indeed embedded in the microstructure, causing slight expansion, which transitions toward disordered graphite regions [72]. In some cases, no change in interlayer spacing is observed, likely due to two reasons: (i) in X-ray diffraction (XRD) characterization, the (002) diffraction peak is broad, and *d*_002_ is typically calculated only at the peak's highest point; and (ii) the space occupied by a small number of sodium atoms may be insufficient to induce an overall change in the microscopic structure [49, 73]. Therefore, the validity of GITT as supporting evidence for the composite model requires further verification.

In contrast to the three-stage model, Cao et al. [59] proposed the "adsorption–intercalation–filling" model (Fig. 3c). This study investigated and analyzed the GITT profiles of carbon materials with varying microstructures. The glucose-derived hard carbon electrode (Glu), dominated by pseudo-graphite domains, displayed the classic three-stage curve, corresponding to the special inflection point at the end of the plateau region discharge curve. In contrast, the Mg glucose-derived hard carbon electrode (Mg-Glu), characterized by a higher concentration of closed pores, maintained an almost constant diffusion coefficient in the plateau region, and the inflection point at the discharge voltage of 0 V disappeared. The explanation for the Mg-Glu sample is that the broad distribution of interlayer spacing and pore sizes results in partial overlapping binding energies, which leads to both intercalation and filling occurring simultaneously during discharging in plateau region. It is crucial to note that alloy-type and conversion-type electrode materials typically exhibit significant decreases in diffusion coefficients during phase transitions, followed by a rapid recovery [74]. Pair distribution function (PDF) data analysis also indicated that as interlayer spacing continues to increase, the intercalation and filling mechanisms become similar, which is attributed to the extended interlayer spacing being very similar to the structure of smaller pores [75].

In short, although the comprehensive mechanisms in HC remain unclear, closed pores are widely recognized as efficient active sites in the plateau region.

### Structures and Parameters of Closed Pores

**Fig. 4:**
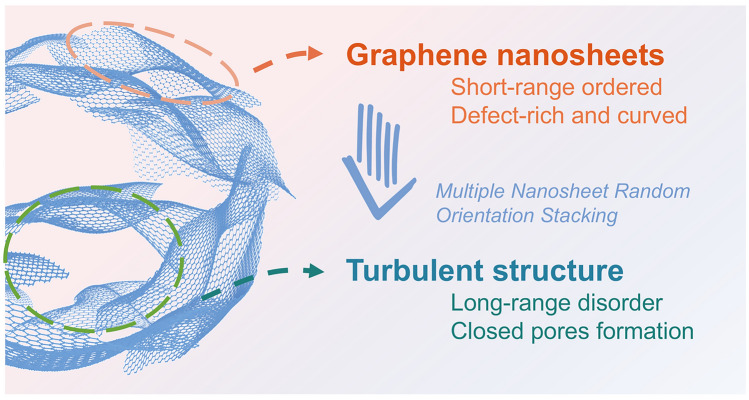
Microscopic illustration of graphene nanosheets (shown within the pink dashed box) and turbulent structures (shown within the green dashed box) in HC materials

Before delving into the process of closed pore formation, it is crucial to first establish a clear understanding of the overall microstructure of HC and the composition of its framework. Currently, the ideal microstructure of HC is broadly accepted as follows (Fig. 4): At a local scale, stripe-like pseudo-graphitic microcrystals are formed by slightly twisted graphene nanosheets arranged in a few-layer stack (typically 2–4 layers), exhibiting short-range order owing to the presence of defects. At a larger scale, these microcrystals, as fundamental structural units, are distributed irregularly, resulting in a turbulent structure that gives rise to both open and closed nanopores [76]. Typically, the primary techniques for studying Na intercalation in the pseudo-graphite region are in situ and ex situ X-ray diffraction (XRD) analysis. XRD patterns are highly sensitive to structural changes in HC materials induced by the insertion of alkali metal ions. They exhibit two characteristic broad diffraction peaks at approximately 24° and 43°, corresponding to the (002) and (100) reflections of disordered carbon's pseudo-graphitic domains. Using Bragg's law and the Debye–Scherrer equation, the average interlayer spacing (*d*_002_), average width (*L*_a_), average thickness (*L*_c_), and stacking number (*n*) of the graphite microcrystals can be calculated [77]. As the fundamental determinants of closed pores, the properties of the crystal structure dictate the specific characteristics of these pores, including the curvature of pore walls, pore entrances, pore channels, and pore size. These pore parameters are temperature-dependent, with open pores being transformed into closed pores at higher temperatures.

#### Pore Walls with Curvature

**Fig. 5:**
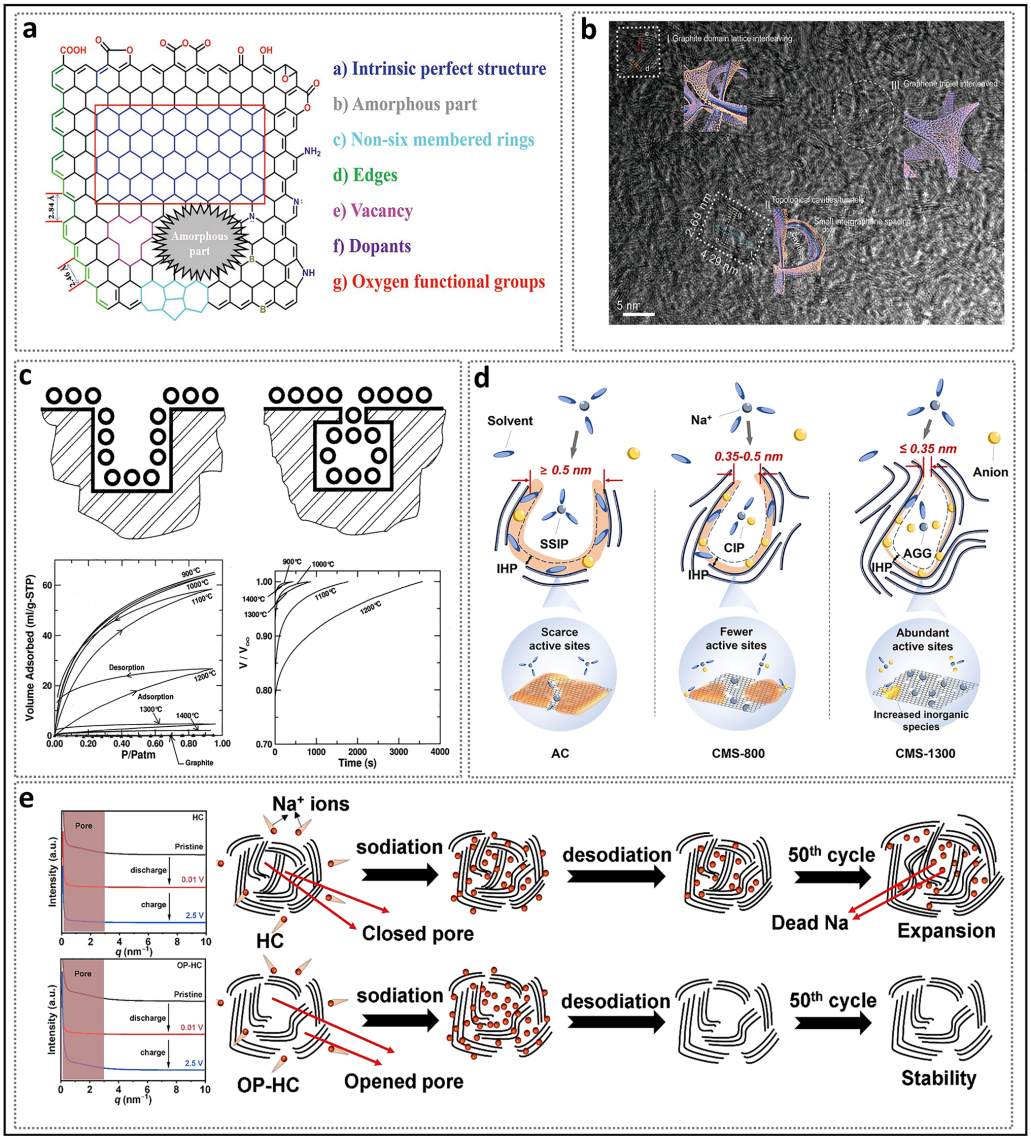
Origin of pore wall curvature and the formation of pore entrances and channels. **a** A typical carbon layer on the carbon surface with different kinds of defects [83]. Copyright 2022, Wiley. **b** HRTEM image of high topological graphitized carbon. Reproduced with permission [87]. Copyright 2023, Wiley. **c** Schematic diagram of open and closed pores, and the adsorption profiles and rates of HC prepared at different temperatures. Reproduced with permission [89]. Copyright 1999, Elsevier. **d** Diagram of pores with different sizes of entrances and their impact on the sodium storage mechanism. Reproduced with permission [90]. Copyright 2025, Springer Nature. **e** SAXS patterns and illustrations of the (de)sodiation process in HC with channels of different sizes. Reproduced with permission [69]. Copyright 2024, The Royal Society of Chemistry

Existing models suggest that curvature plays a crucial role in the formation of HC because it prevents the formation of long-range ordered carbon layers, as seen in soft carbon [78]. The pore wall curvature in the turbulent structure originates from the different hybridization states of the carbon atoms [79, 80]. The planar hexagonal network of graphite layers is composed of *sp*^2^-hybridized carbon atoms with high conductivity, strong van der Waals forces, and an orderly stacking arrangement, making it difficult to bend the well-ordered layered crystal structure. In contrast, HC precursors form a complex and robust cross-linked system due to interactions between heteroatoms, often containing a larger proportion of *sp*^3^ carbon atoms [81]. During high-temperature processes in an inert atmosphere, *sp*^2^ carbon (C=C) shows low reactivity due to the stability of the delocalized graphene structure [82]. On the other hand, *sp*^3^ carbon (C–C) with C–H bonds and weakly stable functional groups is more prone to bond breaking, leading to the formation of dangling bonds related to free radicals, thus inducing carbon atom loss (vacancies), non-hexagonal structures, and inherent defects such as edges (Fig. 5a) [83]. Notably, topological defects like pentagons, heptagons, and their combinations forming Stone–Wales defects exhibit different bond lengths or angles, disrupting the integrity of the sp^2^ carbon conjugated network growth and leading to the bending of carbon layers [54, 84, 85]. Ultimately, the cross-linked system evolves into a turbulent structure of HC with a larger interlayer spacing. It is worth noting that, once the pyrolysis process ends, the curvature of these nanosheets decreases with increasing graphitization [86]. However, even after treatment at elevated temperatures of 2000 °C, topological defects persist, highlighting that cross-winding is an intrinsic property of HC nanosheets (Fig. 5b) [87]. Furthermore, the residual heteroatoms are often located at the edges, referred to as extrinsic defects [88].

#### Pore Entrances and Channels

Unlike the flat graphite domains in soft carbon, the curved nanosheets in HC exhibit considerable advantages in sodium storage. In the turbulent three-dimensional structure, microcrystals, which act as pore walls, can extend in any direction. When two or more microcrystals approach each other at non-parallel angles, the outermost ends (compared to the inner regions) form narrower, contracted pore entrances. A pore structure may sometimes consist of multiple nanosheets, leading to a region with multiple possible entry/exit points for sodium atoms. The micropore closure model proposed by Dahn and others suggests that pores gradually close, as evidenced by a decrease in gas detectability and CO_2_ adsorption rates with increasing heat treatment temperatures (Fig. 5c) [89]. More importantly, this process involves a desorption hysteresis phenomenon, in which the intermediate state of partially closed pore entrances (e.g., ink-bottle-shaped pores, slit-shaped pores) emerges and subsequently disappears. While parameters such as shape and size also influence the diffusion of electrolytes and gases within pore structures, the size of pore entrances is undoubtedly a critical indicator in pore-type transitions. Yang et al. [90] recently pointed out that when the pore mouth is fully open (> 0.5 nm), both N_2_ and CO_2_ molecular probes can be accessible (Fig. 5d); when the pore entrance is between 0.35 nm and 0.5 nm, N_2_ cannot enter, but CO_2_ molecular probes can still enter; when the pore mouth is smaller than 0.35 nm, CO_2_ molecular probes cannot be accommodated. Based on these detailed data, the definition method of closed pores has been further refined.

Research has demonstrated that although the concentration of closed pores typically remains stable, continued heating to a certain temperature results in partial accessibility of the nanopores, leading to an anomalous decrease in plateau capacity [46]. This further implies that only over-graphitized closed pores are almost entirely sealed, whereas the ideal closed pores, as typically anticipated, are partially open. One possibility is the excessive contraction of closed pores, but considering the difficulty of graphite formation in hard carbon and the repulsive forces between the nanosheets, this is very challenging. Another reasonable explanation is to view this phenomenon in terms of the blockage of the diffusion path in the pore channels [91], as the pores are not isolated but interconnected. Additionally, the poor rate performance of hard carbon materials reflects the presence of high-crystal graphite channels. Pore channels refer to regions surrounding the pore gaps that are not occupied by other pore walls, with both the length and thickness of the pore walls being critical factors [92]. If the average L_a_ continues to increase, larger nanosheets will compress the pore channel space [50]. Additionally, the porosity and density of the turbulent structure channels can be characterized by *d*_002_ and *L*_c_. When *d*_002_ is large and *L*_c_ is small, graphene nanosheets tend to distribute non-parallel rather than stack. At this stage, the pore channels surrounding the graphite walls allow sodium ions to approach. In contrast, when *d*_002_ is small and *L*_c_ is large, dense stacking of pore walls accompanies the pore structure, effectively shielding the pore gaps. Even if the pore structure is filled during the initial charge–discharge cycles, poor structural stability may lead to errors, causing the pores to become more blocked and resulting in irreversible capacity loss. Yang et al. [69] investigated the evolution of pore structures during the first charge–discharge cycle in samples with different *d*_002_ using SAXS (Fig. 5e). Their findings revealed that the slope of the OP-HC for samples with a larger channel could be consistently maintained, demonstrating high reversibility. In contrast, the slope of the HC with smaller channel disappeared. At a deeper level, this can be attributed to sodium atoms filling excessively closed pores, leading to the formation of irreversibly trapped dead sodium that cannot be released [45].

**Fig. 6:**
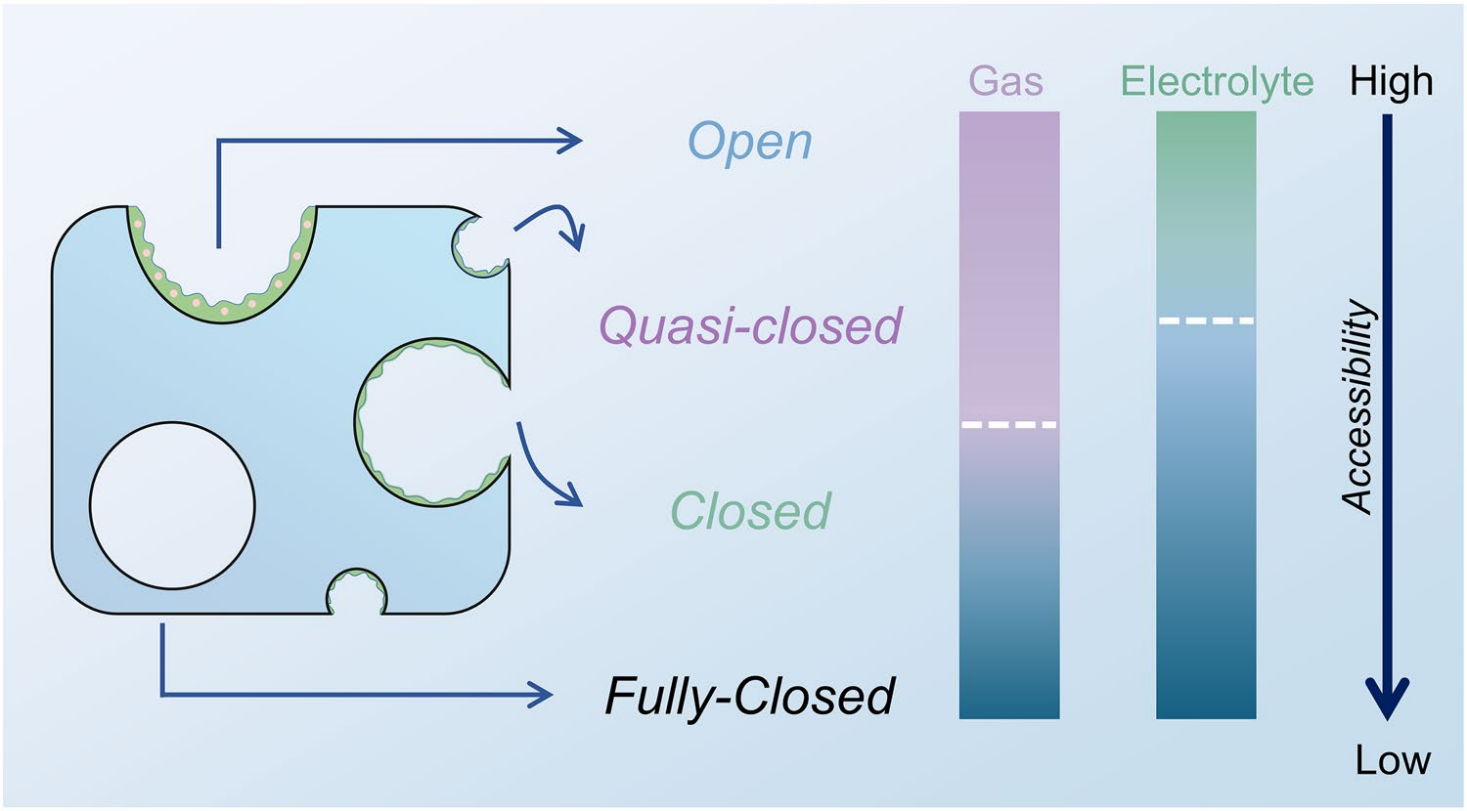
Schematic diagram of pore classification based on fluid accessibility. The white dashed line represents the boundary, with purple and green indicating gas and electrolyte accessibility, respectively, and blue indicating inaccessibility

Based on the aforementioned research findings, we propose the concepts of "quasi-closed pores," "closed pores," and "fully closed pores" (Fig. 6) to elucidate the sources of high ICE and long plateau capacity in HC, as well as to explain the phenomena observed in adsorption–desorption experiments. Specifically:

i. The HC materials prepared at low temperatures are almost entirely open pores, with a relatively large distance between the nanosheets that allows contact with external gases and complete exposure to the external electrolyte. The open pores only contribute to the slope capacity and result in very low ICE and platform capacity.
ii. If the heat treatment temperature is moderate, the gaps between the nanosheets of the obtained HC material partially shrink, forming quasi-closed pores (including ultramicropores). The bent nanosheets of the quasi-closed pores maintain some space between them, allowing gas detection, which can partially reflect the overall size and shape of the closed pores. As the carbonization temperature increases, the crystallinity of HC continuously enhances. The closed pores first become undetectable to N_2_ with a larger kinetic diameter, followed by the disappearance of the smaller CO_2_ signal. Some electrolyte will be blocked, corresponding to some slope capacity being converted into platform capacity.
iii. At higher preparation temperatures, the closed pores are formed through the tight interlocking of graphite microcrystals, leaving only narrow gaps. At this stage, gas entry is prohibited (usually referring to N_2_), and solvent molecules are also highly restricted. These ideal closed pores effectively utilize a large amount of irreversible capacity and significantly contribute to extending the platform capacity.
iv. When the carbonization temperature exceeds the critical threshold (depending on the precursor), some of the closed pores transite into fully closed pores, becoming even inaccessible to the electrolyte, and the platform capacity decreases instead [93].

We believe that the classification method based on pore gaps, incorporating quasi-closed pores and fully closed pores, can effectively and logically explain the correlation between pore structure and mechanisms, while also facilitating future research on fully-closed pores and specific ultramicropores. However, this classification method considers a broader range of pore characteristics, some of which cannot be quantified, which may introduce additional complexity into the discussion. In practice, during the preparation of HC, most of the pores obtained are typically closed pores; therefore, the analysis can be simplified accordingly.

#### Pore Size and Volume

The nanosheet crystal size of the pore walls to some extent determines the pore size and pore volume [94]. It is well known that the *L*_a_ of the crystals grown can vary significantly depending on the material source and the preparation process (such as changes in final temperature, heating rate, and pre/post-treatment). Typically, for a pore region, the surrounding longer pore walls can expand the occupied space, thereby increasing the pore size and pore volume. For carbon materials that use closed pore engineering, most completely closed pores cannot have their pore size distribution measured like quasi-closed pores. Therefore, the average pore size, *R*, is an important piece of information that must be obtained for closed pores. It should be noted that due to the significant irregularity of pore structure shapes, they are usually assumed to be spherical for calculation and reference, even though ideal spherical closed pores are almost impossible to achieve.

**Fig. 7:**
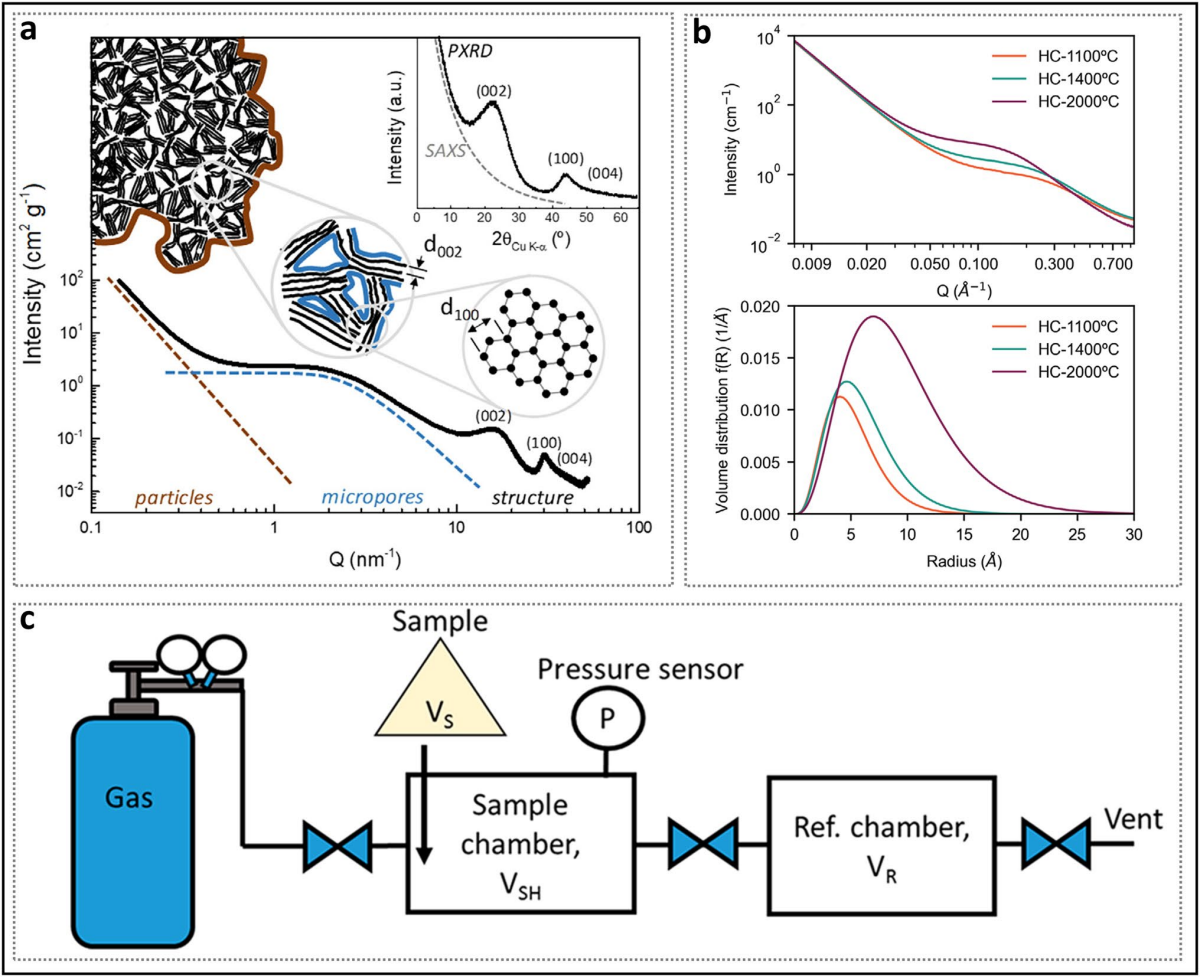
Some parameters for characterizing pores. **a** Full-range plot of scattering patterns from SAXS to PXRD. Reproduced with permission [79]. Copyright 2019, Elsevier. **b** SAXS profiles of HC prepared at different temperatures and the pore size distribution obtained from modeling the SAXS data. Reproduced with permission [95]. Copyright 2023, Wiley. **c** Schematic of a typical helium pycnometer. Reproduced with permission [96]. Copyright 2019, American Chemical Society

Compared to techniques such as mercury intrusion and nitrogen adsorption, SAXS is a crucial method for obtaining the quantitative properties of the average pore size in nanopore systems (Fig. 7a) [79]. When a beam of highly focused X-rays is directed at the material surface, scattering occurs within 5° of the incident beam if nanoscale particles or regions with uneven electron density are present. X-rays possess superior detection capabilities for both open and closed pores, and the resulting measurements are highly accurate. In SAXS, the scattering intensity, *I*_*q*_, as the vertical axis, generally decreases with increasing 2*θ* and is closely related to the size and shape of the HC pore structure. This variation is manifested in the small-angle scattering intensity curve, where the horizontal axis is represented by the wave vector *q* (Å^−1^), as defined in Eq. 1. The shoulder peak near 0.1 Å^−1^ corresponds to the presence of periodically arranged nanopores. The more pronounced the shoulder peak, the higher the nanopore concentration (Fig. 7b) [95]. As the heat treatment temperature increases, the shoulder peak generally shifts toward lower *q* vectors, indicating the formation of larger-scale nanopores [60]. The Guinier theorem states that at small *q* vectors (*qR*_*g*_ < 1), the scattering distribution can be approximated by Eq. 2. When the Guinier approximation holds, the ln*I*_q_-*q*^2^ curve becomes linear, and the slope of the line can be used to calculate the gyration radius, *R*_g_. When calculating the pore structure radius, *R*_g_ needs to be further processed using the spherical particle conversion formula (Eq. 3).

1 $$q = \frac{4\pi \sin \theta }{\lambda }$$

where *λ* = 1.541 Å is the X-ray wavelength of Cu K_*α*_ and 2*θ* is the scattering angle.

2 $$I_{q} = I_{0} e^{{ - \frac{{R_{{\text{g}}}^{2} }}{3}q^{2} }}$$

In the equation, *I*_0_ is the scattering intensity at *q* = 0, which can be obtained by extrapolation, and *R*_g_ is the gyration radius, representing the root-mean-square distance of each electron from its center of mass.

3 $$R = \sqrt{\frac{5}{3}} R_{{\text{g}}}$$

where *R* refers to the average pore diameter.

Numerous enhanced SAXS models and approximation formulas have been proposed to more precisely calculate the average pore diameter [61, 95]. Additionally, crucial statistical information, such as the pore size distribution and specific surface area (SSA) of all pores, can also be obtained, which plays a pivotal role in guiding pore design. It is important to note that, to determine the SSA of closed pores, the calculated value from simulations must be subtracted from the *S*_BET_ measured by N_2_ adsorption. However, due to the use of two distinct characterization methods, the resulting data may contain a certain degree of error.

For pore volume, the He gas displacement method (or He pycnometer method), based on Archimedes' principle, is commonly employed to measure pore volume (Fig. 7c) [96]. The He gas used in the measurement has an extremely small kinetic diameter (2.6 Å) at room temperature, and its penetration depth is sufficient to access all open pores. The volume occupied by the carbon material (*V*_s_) is calculated via a pressure balancing process, in which the measurement chamber is connected to a reference chamber. The particle density (*ρ*_s_) of the sample is determined using Eq. 4. Subsequently, the closed pore volume (*V*_closed pores_) is calculated using Eq. 5 and 2.26 g cm^−3^ (the true density of ideal layered graphite without any pore structure).

4 $$\rho_{{\text{s}}} = \frac{{m_{{\text{s}}} }}{{V_{{\text{s}}} }}$$

In the equation, *ρ*_s_ is the particle density of the sample, which refers to the actual mass of solid material per unit volume in an absolutely dense state. *m*_s_ is the sample mass, and *V*_s_ is the sample volume.

5 $$V_{{{\text{closedpores}}}} = \frac{1}{{\rho_{{\text{s}}} }} - \frac{1}{2.26}$$

Here, *V*_closed pores_ represents the closed pore volume of the sample.

However, the accuracy and applicability of the He gas displacement method require verification, as the calculation of the *V*_closed pores_ varies significantly across different studies. Even within a single study, establishing a clear correlation between pore volume and plateau capacity remains challenging. This discrepancy may arise from the choice of reference structure, as significant differences exist in lattice parameters (such as interlayer spacing) between ideal graphite and HC. In addition, the unclear definition of previously closed pores and the complexity of the sodium storage mechanism in closed pores have deepened the inconsistency of the results. Therefore, the key role of volume parameters still requires further investigation. Recently, Toney et al. [95] innovatively combined SAXS and particle density to calculate the volume fraction of pores, thereby circumventing the need for the true density of graphite.

### Sodium Storage Mechanism of Closed Pores

Closed pores and open pores have distinctly different sodium storage mechanisms, which are reflected in several aspects, including the insertion of sodium ions during electrolyte entry, the stable state during sodium-ion storage, and the specific process of sodium-ion filling.

#### Insertion of Sodium Ions

**Fig. 8:**
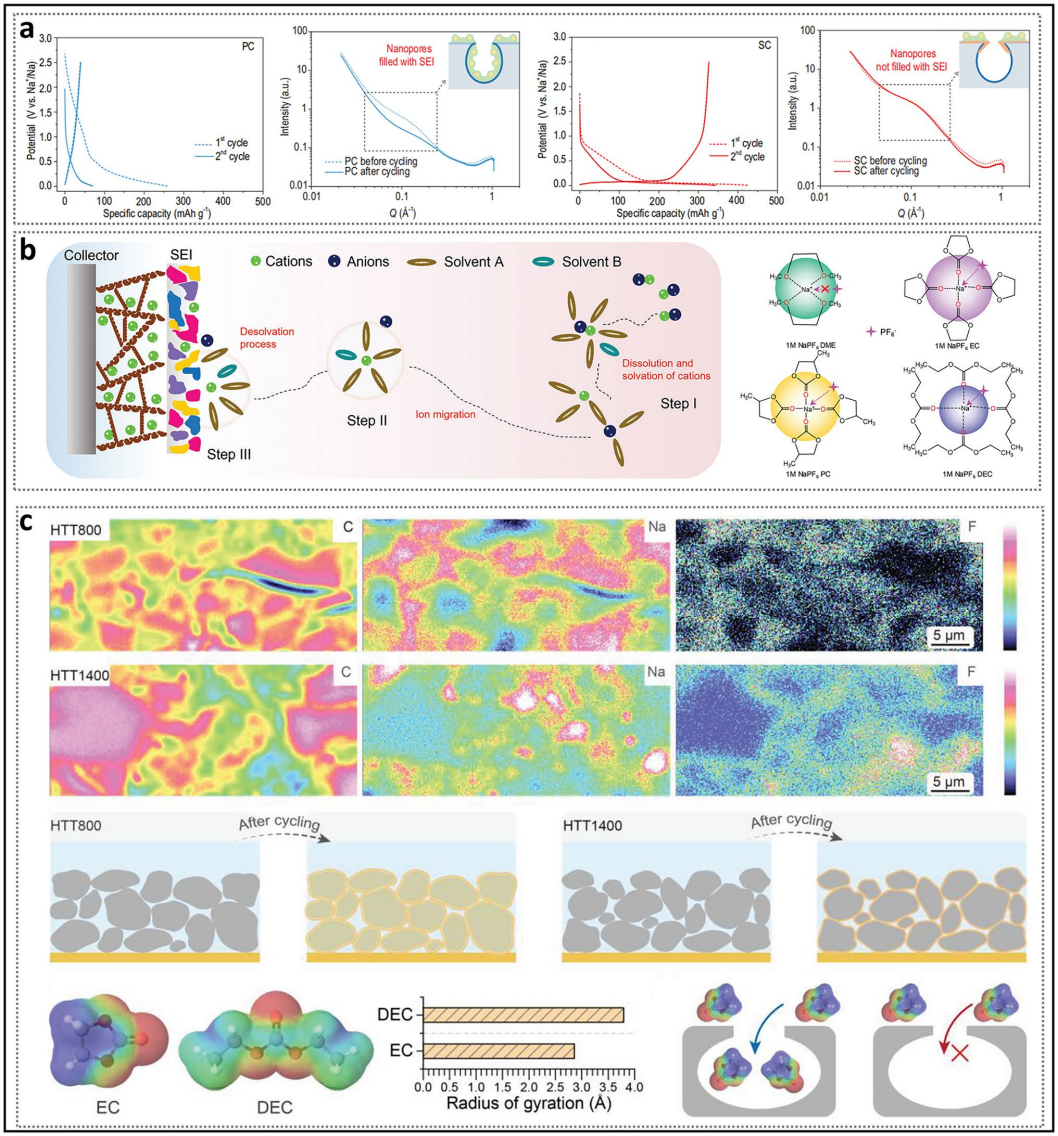
Solvation process and the formation of the SEI. **a** Charge/discharge curves for the first two cycles and the SAXS patterns before and after five full cycles of HC with different pore structures. Reproduced with permission [61]. Copyright 2022, Oxford Academic. **b** Illustration of the impact of coordination structure and solvation process on electrochemical performance. Reproduced with permission [98]. Copyright 2022, Wiley. **c** EPMA characterization of HTT800 and HTT1400, and the performance of the solvation structure with size determined through theoretical calculations when passing through closed pores. Reproduced with permission [65]. Copyright 2024, Wiley

SAXS analysis suggests that the increase in ICE results from the spatial confinement of ideal closed pore gaps. This confinement impedes the formation of undesirable SEI on the inner walls of the nanopores during the first cycle (Fig. 8a) [61]. However, SEI alone cannot account for all experimental observations within its inherent logical framework. For instance, samples with identical SBET values show considerable differences in ICE. The key active sites within the closed pores, which contribute to the reversible plateau capacity, differ from those in open pores since they must first traverse narrow pore necks. Consequently, a growing number of researchers have acknowledged the pivotal role of the solvation process and have sought to elucidate the solvation structure of the electrolyte [97]. The solvation process describes the dissolution of sodium ions in an organic solvent to form a solvation structure, followed by migration and desolvation at the electrolyte–electrode interface. In the solvation structure, the sodium ion is surrounded by two solvation shells, within which solvent molecules and anions interact with the sodium ion through electrostatic forces and coordination bonds (Fig. 8b) [98]. Taking ultramicropores as an example, these micropores can act as molecular sieves or ion sieves without compromising ion diffusion capabilities [99–101]. Similarly, other larger closed pores can achieve comparable sieving effects owing to the presence of pore entrances [102, 103]. In the study by Zhang et al. [65], electron-probe X-ray microanalysis (EPMA) revealed the signal intensities of various elements in the cross-sectional views of HC electrodes at different temperatures (Fig. 8c). In HTT800 (HC prepared at a heat treatment temperature of 800 °C, hereinafter referred to as the same), the distribution of carbon and sodium signals was more similar, attributed to the permeation of solvent molecules, which led to the formation of substantial amounts of SEI on both the inner and outer surfaces of the particles. In contrast, the sodium signal in HTT1400 primarily appears in regions with low carbon signal intensity, indicating that sodium loss mainly occurs on the outer surface of the particles. Aside from the pore entrance, the size of the solvation structure is a crucial factor influencing the sodium insertion process. Specifically, solvent molecules, such as EC with a minimum dynamic diameter of 5.7 Å, would be geometrically restricted by the closed pore entrances. Bare Na^+^ (with an ionic radius of 1.02 Å), lacking a solvation shell, is sufficiently small to freely enter and exit the region. Given that the kinetic diameter of N_2_ is smaller than that of solvent molecules, this explains why it can detect quasi-closed pores.

**Fig. 9:**
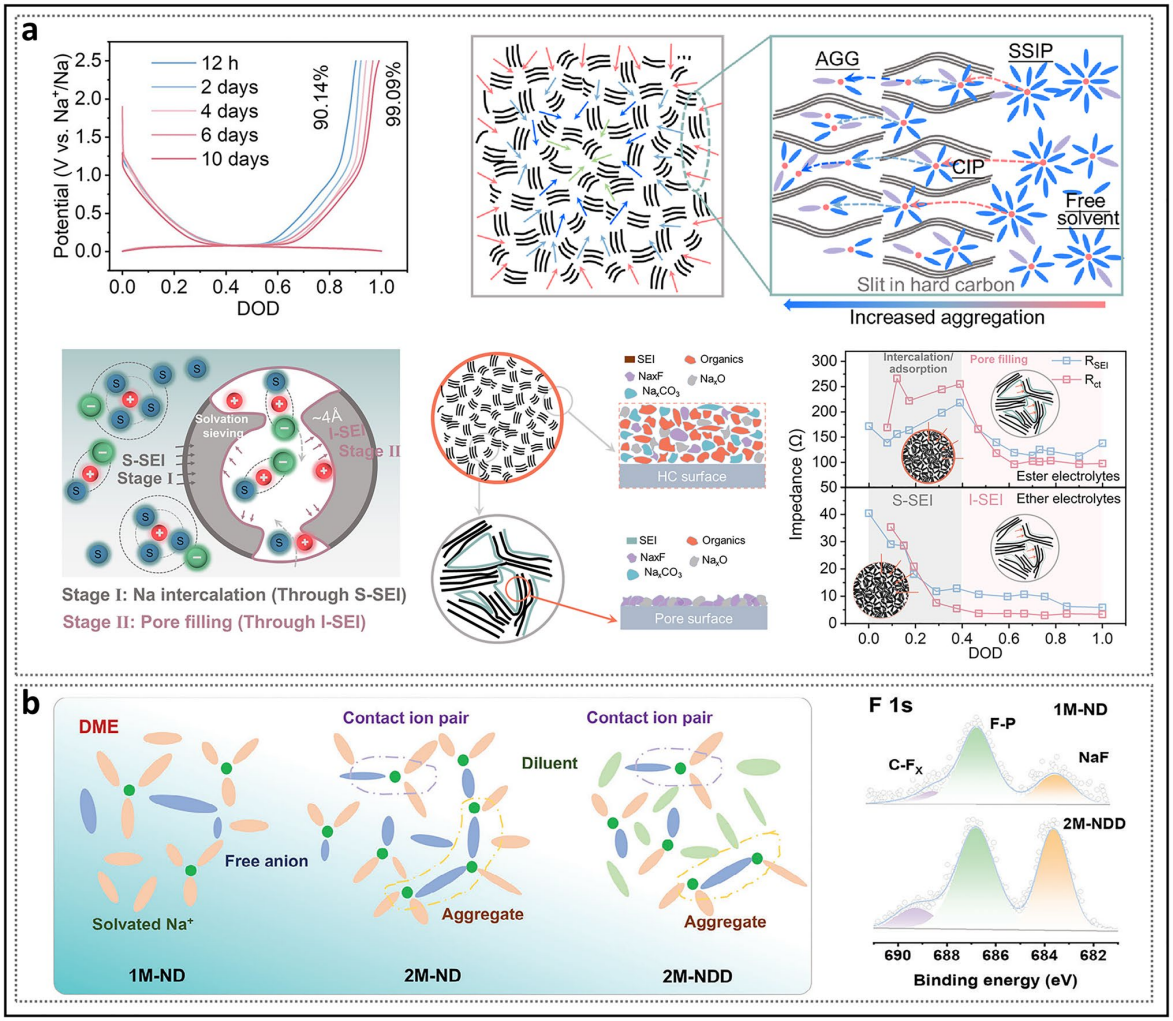
Analysis of the solvated structure. **a** Illustration of the pre-desolvation of the electrolyte on HC, along with the schematic diagram of the dual-layer SEI model and its distinction. Reproduced with permission [104]. Copyright 2024, Springer Nature. **b** Brief modeling of the solvated structures for electrolytes with different concentrations and the corresponding F 1s spectra. Reproduced with permission [35]. Copyright 2024, Wiley

Considering that more than one solvent molecule is bound to the sodium ion, the desolvation process may occur gradually until the solvation structure is able to pass through the pore entrances [64]. Zhou et al. [104] presented a clear depiction of the desolvation process in closed pores (Fig. 9a). In their experiment, they found that aging the half-cell without compromising stability or cycle life could significantly enhance the ICE (99.09%, in ether-based electrolytes) and reduce interface impedance. This time-dependent behavior arises from the spontaneous and gradual wetting of the electrolyte within the pores. This process is explained by the fact that, when solvent-separated ion pairs (SSIPs) pass through size-constrained nanopores, the solvent shell gradually dissociates, transforming into contact ion pairs (CIPs) and ultimately aggregates (AGGs). Notably, bare Na^+^ ions are not formed at the end, indicating that solvent molecules continue to enter the pores. Based on this, they rationally proposed a dual SEI model, which provides further insight into the sources of irreversible Na loss. The model suggests that a S-SEI forms on the surface of the carbon layer, while a thinner I-SEI, with a higher proportion of inorganic components, forms inside the nanopores. These two types of SEI exhibit significant differences in resistance during the discharge process, reflecting distinct ion transport mechanisms between them. Based on this, adjusting the solvation structure is an effective strategy for promoting the formation of a good SEI [105]. In conventional electrolyte concentrations, the primary solvation sheath consists predominantly of SSIPs. At this stage, the efficiency of the anion reduction reaction is relatively low, resulting in the generation of a SEI that is rich in organic components. To form more CIPs and ACGs, the key is to promote the inclusion of anions into the primary solvation sheath. For instance, Fan et al. [35] reported a strategy in which high-concentration electrolyte (2.0 M NaPF_6_) and a diluent (1,3-dioxolane) were incorporated into an ether-based electrolyte, resulting in a substantial enhancement in the cycling stability of HC (Fig. 9b). Thinner, more uniform, and NaF-rich SEI are achieved with an optimized solvation structure that contains fewer solvent molecules. At the same time, the introduction of a diluent effectively balances the high viscosity caused by high salt concentration.

#### Stable State

The correlation between the steady state of closed pore structures and plateau capacity has long been a central focus of research on HC. Magic angle spinning solid-state NMR (MAS ssNMR) is widely used as a powerful technique for investigating this issue [106, 107]. The rotor is rapidly spun at an angle of 54.7° relative to the direction of the static magnetic field, averaging dipolar and anisotropic interactions, thus significantly narrowing the solid-state NMR spectra for high-resolution analysis. In general, the peaks observed in HC can be broadly classified into two categories. One type appears around 0 ppm, attributed to the peak of diparamagnetic sodium ion. This peak typically arises from the superposition of multiple Lorentzian components. It originates from sodium salts in the electrolyte, Na atoms near the carbon layer, and Na by-products in the SEI [108]. Another type is the quasi-metallic peak in the closed pores, which arises from the interaction between the nuclear spin and the unpaired Na 2s electrons at the Fermi level of the conduction band, resulting in a Knight shift.

**Fig. 10:**
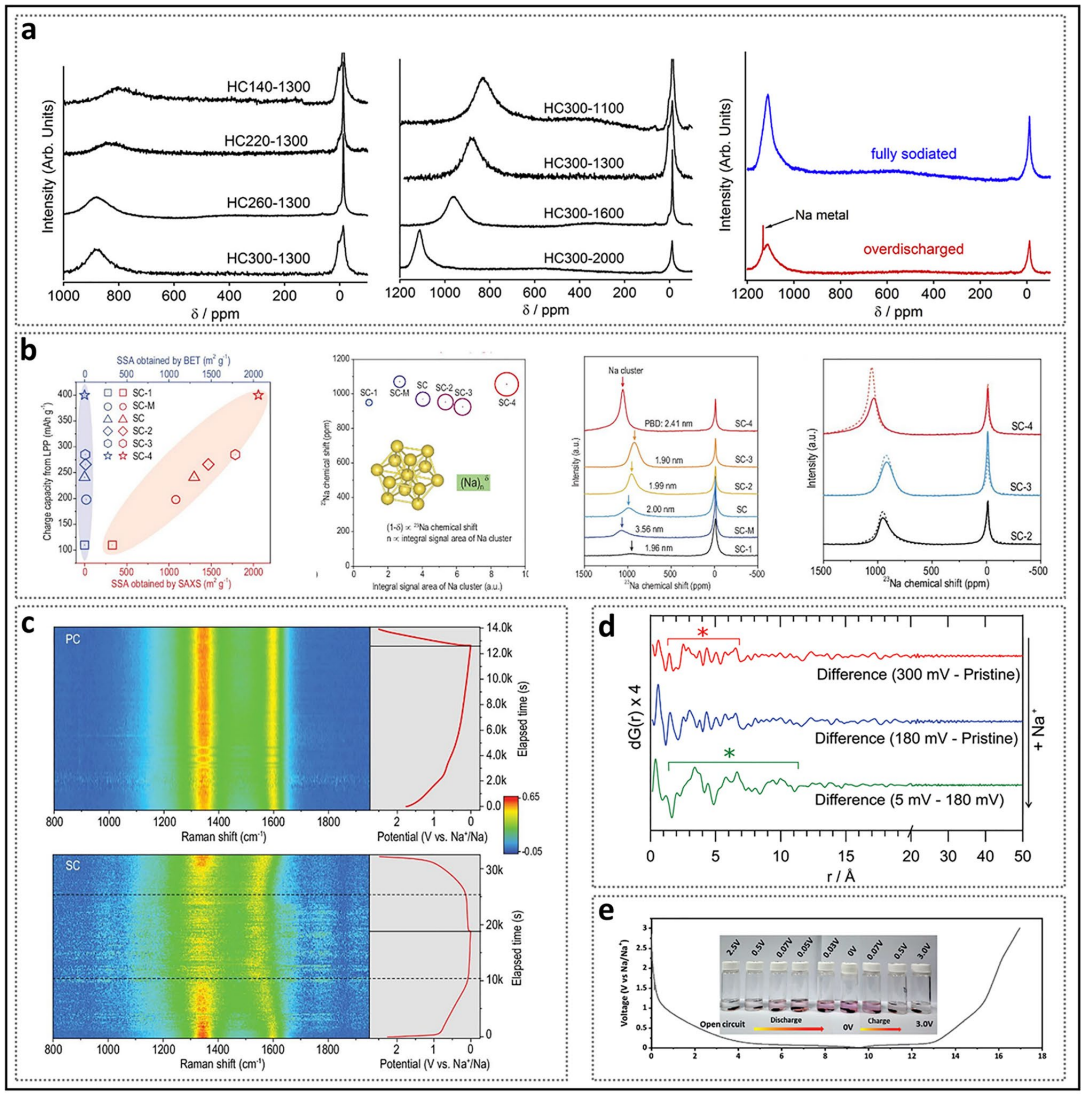
Stable state of sodium filling in closed pores. **a** ^23^Na NMR spectra of fully sodiated HC samples prepared at different dehydration temperatures and carbonization temperatures. Reproduced with permission [109]. Copyright 2019, Elsevier. **b** Correlation between the pore parameters and physicochemical properties of sodium clusters. **c** *Operando* Raman spectra of HC with different pore structures during the first charge/discharge. **b–c** Reproduced with permission [61]. Copyright 2022, Oxford Academic. **d** Difference PDFs of HC anodes at various states of charge. Reproduced with permission [54]. Copyright 2016, The Royal Society of Chemistry. **e** Color changes of ethanol containing 1% phenolphthalein after reaction with HC at different potentials. Reproduced with permission [55]. Copyright 2021, Wiley

The Ishida group investigated a series of HC subjected to varying dehydration and annealing temperatures (Fig. 10a) [109]. As the treatment temperature increased, the quasi-metallic peak progressively shifted to higher frequencies, reaching a maximum of 1114 ppm, a value that is in close proximity to the 1130 ppm observed for metallic sodium. This suggests that larger sodium clusters may form in closed pores with larger pore sizes. Sodium deposition was observed only during over-discharge, exhibiting a sharp peak distinct from the quasi-metallic peak. They also provided an explanation for the absence of quasi-metallic sodium in certain HC materials with closed pores in prior studies, attributing it to the introduction of the electrolyte additive fluoroethylene carbonate (FEC), which caused incomplete sodiation of the HC at the cutoff voltage [52, 53]. Yang et al. [61] conducted a comprehensive study on the changes in quasi-metallic sodium (Fig. 10b). They converted the open pores of four microporous carbons and one mesoporous carbon into closed pores, resulting in SC-X (with higher SSA corresponding to larger numbers) and SC-M. The results indicated that the low-voltage plateau capacity of these carbon materials was positively correlated with SSA (measured by SAXS) and the integral signal area in the ssNMR spectrum, which reflects the number of sodium atoms aggregated per unit mass of SC. Therefore, porous carbon precursors with a larger SSA can create more favorable environments for sodium cluster formation after the closure of open pores. Furthermore, the average pore size of the pore structure significantly influences the number of sodium atoms and their metallic character aggregated in individual quasi-closed pore regions, leading to observable changes in chemical shifts. Although the chemical shifts were similar, the plateau capacity of SC-M, derived from mesoporous carbon, was significantly different from that of SC-4. However, SC-4, with the largest plateau capacity and an average pore size of 2.41 nm, exhibited poor cycling stability, accompanied by a substantial decrease in the integrated ^23^Na signal area, indicating that sodium clusters with excessive metal content demonstrate poor reversibility during cycling. Additionally, in two studies by Stratford et al., synchrotron X-ray total scattering pair distribution function (PDF) analysis was employed to identify the local atomic environment of sodium storage in HC. Sodium clusters were observed in multiple carbon materials at approximately 1.0 nm and in the 1.3–1.5 nm range (Fig. 10d), which required larger closed pore sizes to accommodate them [54, 75]. Smaller closed pore (ultramicropores) may hinder the close packing of sodium atoms, making it difficult for sodium clusters to form and expand, and thus traces of quasi-metallic sodium become more difficult to detect [100].

HC exhibits two typical characteristic peaks in Raman spectra, which can aid in confirming the steady-state filling condition [110]. The G-band at 1580 cm^−1^ arises from the E_2g_ stretching vibration mode of *sp*^2^ carbon bonds in rings and chains, and is indicative of the degree of graphitization. In contrast, the D-band at 1350 cm^−1^ is caused by the A_1g_ breathing vibration mode of *sp*^2^ carbon rings, which necessitates the presence of disordered defects in the material and does not appear in perfect graphite. The intensity ratio of the D and G peaks (*I*_D_/*I*_G_) is a common method for evaluating the disorder in carbon materials and is used to establish the correlation between the number of defects and the slope capacity. In situ Raman spectroscopy can characterize the reversible formation of quasi-metallic sodium clusters and analyze the electron transfer process during their formation (Fig. 10c). During the discharge process in the slope region, a significant reversible redshift (decrease in wavenumber) was observed in the G-band, indicating the occurrence of capacitive Na-ion storage. At this stage, Na-C interactions dominate, and electrons primarily transfer to the graphene nanosheets, occupying the π* anti-bonding band, while sodium atoms are stored in a quasi-metallic ionic state [111]. In the plateau region, the G-band remains nearly unchanged, as the electrons do not further occupy the π* anti-bonding band of the carbon layers but gradually transfer to the sodium ions, thereby establishing a dominant trend of Na-Na interactions [55]. The repulsion between sodium ions, acting as electron acceptors, gradually decreases with the reduction of charge. This leads to the accumulation of most sodium ions in clusters with delocalized electrons, thereby forming additional metallic Na-Na quasi-metallic bonds [112]. Meanwhile, the D-band almost disappears, indicating that defects on the carbon surface are covered and suppressed by the adsorbed sodium ions or quasi-metallic sodium.

In addition, HC containing quasi-metallic sodium clusters can induce a unique color change in phenolphthalein ethanol solution, providing a visual indication of the presence of the pore filling mechanism (Fig. 10e) [55]. During discharge, electrodes with a sufficiently high degree of sodiation in the closed pores exhibit partial metallic properties. The protonic solvent CH_3_CH_2_OH undergoes mild redox reactions with these pores, generating CH_3_CH_2_ONa, which causes the pH-sensitive phenolphthalein solution to gradually turn red. At the charging stage, the color gradually returns to its original state, confirming the reversible formation of quasi-metallic sodium in the HC.

#### Process of Filling Method

**Fig. 11:**
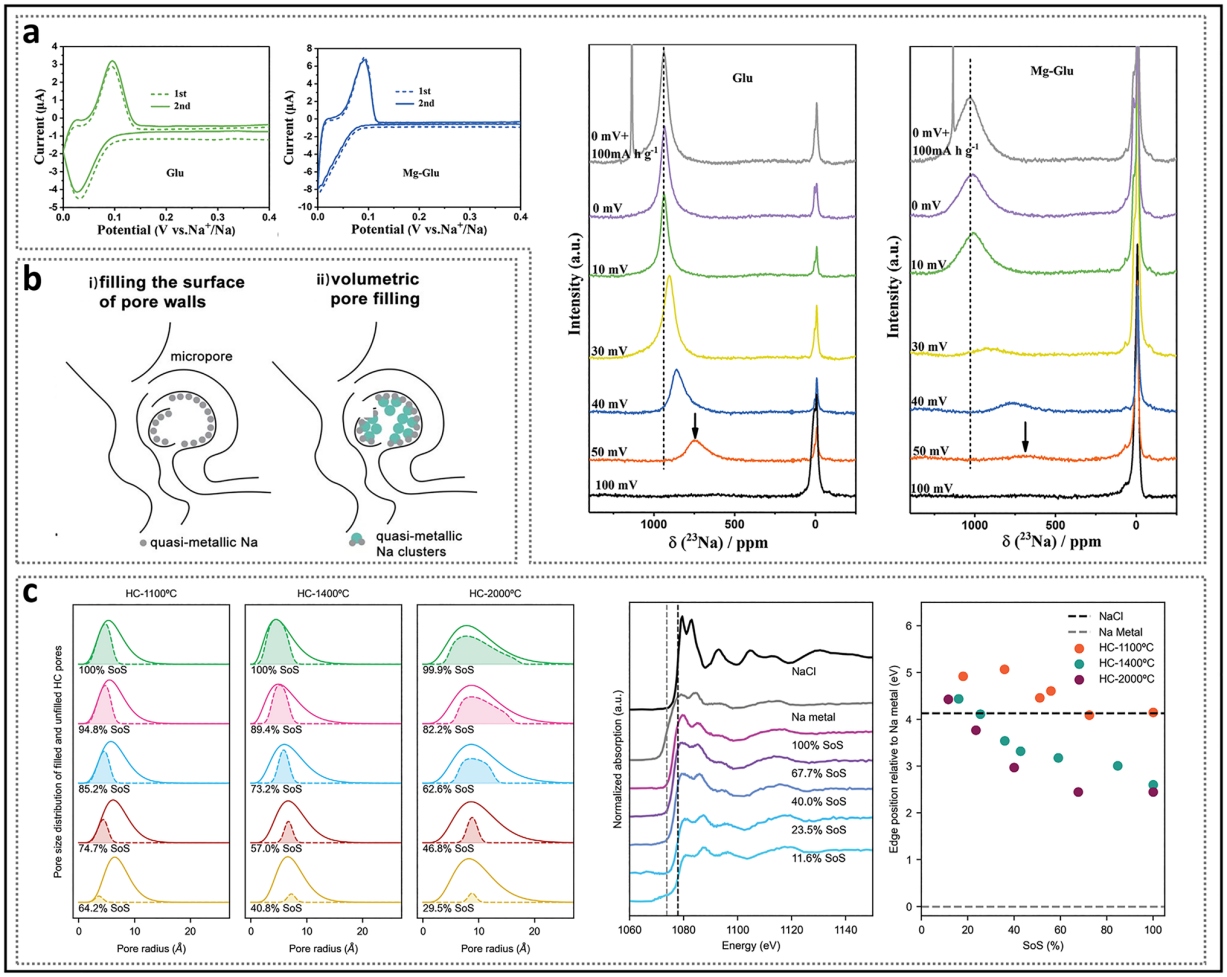
Behavior of sodium filling in closed pores. **a** CV curves of different structured HC electrodes at the voltage window of 1.0–0.002 V and ex situ ^23^Na MAS NMR spectra at different discharge states. Reproduced with permission [59]. Copyright 2022, Wiley. **b** Storage mechanism of sodium within the pores. Reproduced with permission [117]. Copyright 2024, The Royal Society of Chemistry. **c** Pore size distribution and oxidation state of HC prepared at different temperatures under varying degrees of sodium intercalation. Reproduced with permission [95]. Copyright 2023, Wiley

In addition to stable state of filling, the process and uniformity of filling are also crucial aspects that require thorough understanding [113]. In the experiment conducted by Cao et al. [59], two HC materials with different closed pore characteristics, Glu and Mg-Glu, were compared (Fig. 11a). CV and ex situ ^23^Na MAS ssNMR spectra showed that redox peaks and quasi-metallic sodium appeared earlier during discharge in the Glu electrode, which had a slightly lower plateau capacity. The sharp shape of the quasi-metallic peak was attributed to the presence of fewer micropores, which resulted in a more uniform chemical environment. The Mg-Glu, which contained abundant nanopores, exhibited delayed redox peaks and the formation of quasi-metallic sodium. Additionally, its reduction peak failed to form a distinct peak shape. Moreover, the quasi-metallic peak exhibited a broad shape, mainly due to the variation in the chemical environment caused by the diverse micropore dimensions, which led to the formation of clusters with varying scales. Density functional theory (DFT) calculations indicated that the nonlinear relationship between plateau capacity and pore size of closed pores arises from two different sodium storage mechanisms in micropores: (i) localized sodium ions adsorbed by defects and ii) the formation of clusters [73, 114–116]. The significant difference is that, compared to open pores, the chemical environment of defects in closed pores undergoes a substantial change, ultimately leading to a shift in the sodium storage mechanism. The presence of restricted hole entrances can maximize the retention of reversible active sites by preventing defects from interfacial passivation [90]. Specifically, in large closed pores with a low defect density, the nucleation of Na^+^ ions is hindered. The ssNMR experimental results also support this view, indicating that the filling process occurs in two steps: adsorption onto the pore walls and the formation of clusters at positions farther from the pore walls (Fig. 11b) [117]. Toney et al. [95] observed that as the pore size increased, the plateau region shifted to deeper discharge depths. They systematically analyzed the pore size distribution and the oxidation state of the filling using SAXS and X-ray absorption near-edge structure (XANES) (Fig. 11c). At the lowest temperature, HC-1100 predominantly filled smaller pores while retaining its ionic nature throughout the process. This demonstrated that small-pore closed pores exhibit the strongest defect adsorption, promoting a uniform distribution of sodium ions on the pore walls. Based on this, the delayed emergence of the plateau region was attributed to defect healing at elevated temperatures, rather than an increase in pore size. For HC-1400 and HC-2000 at elevated temperatures, the pores with the largest average pore sizes were filled first, while other pores were either filled unevenly or remained unfilled. In particularly, for HC-2000, even pores with average pore sizes were not fully filled, which may be attributed to partial blockage of the pore channels.

### Formation Process of Closed Pore and Possible Issues

**Fig. 12:**
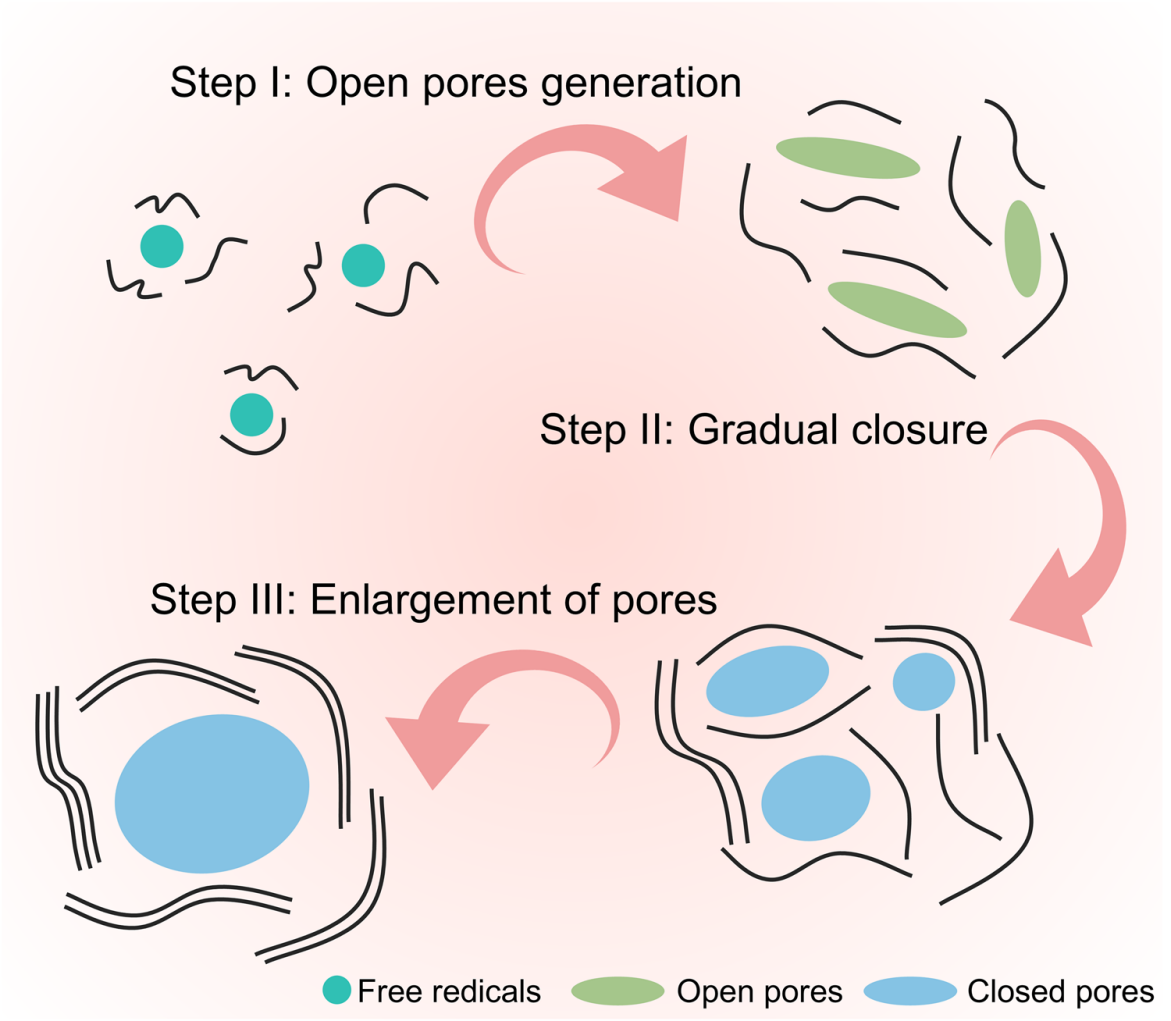
Schematic diagram of the formation of open and closed pores during the conversion of the precursor to HC

Clarifying the specific process of closed pore formation is of crucial significance for guiding structural optimization and design [18]. In simple terms, HC with abundant closed pores is prepared through the carbonization (broadly referring to the entire heating process) of precursors with low graphitability in an inert atmosphere. High-temperature treatment is essential for the formation of closed pores, but it must be conducted within an appropriate temperature range. At moderate temperatures, the formation of graphite microcrystalline stripes with intermediate lengths and increased interlayer spacing is considered a prerequisite for the construction of well-defined closed pores. If a lower sintering temperature (< 700 °C) is employed, the resulting HC inevitably retains a high concentration of irreversible structural defects, hindering the practical applicability of HC. To allow for sodium-ion accessible diffusion channels and prevent the appearance of fully closed pores, the temperature is generally maintained below 1600 °C. The ideal closed pore formation process includes three stages (Fig. 12): the generation of open pores (thermal decomposition releasing free radicals), the development of bent pore walls (defect repair), and the evolution of pore structures (pore merging and shrinkage).

#### Process of Closed Pore Formation

**Fig. 13:**
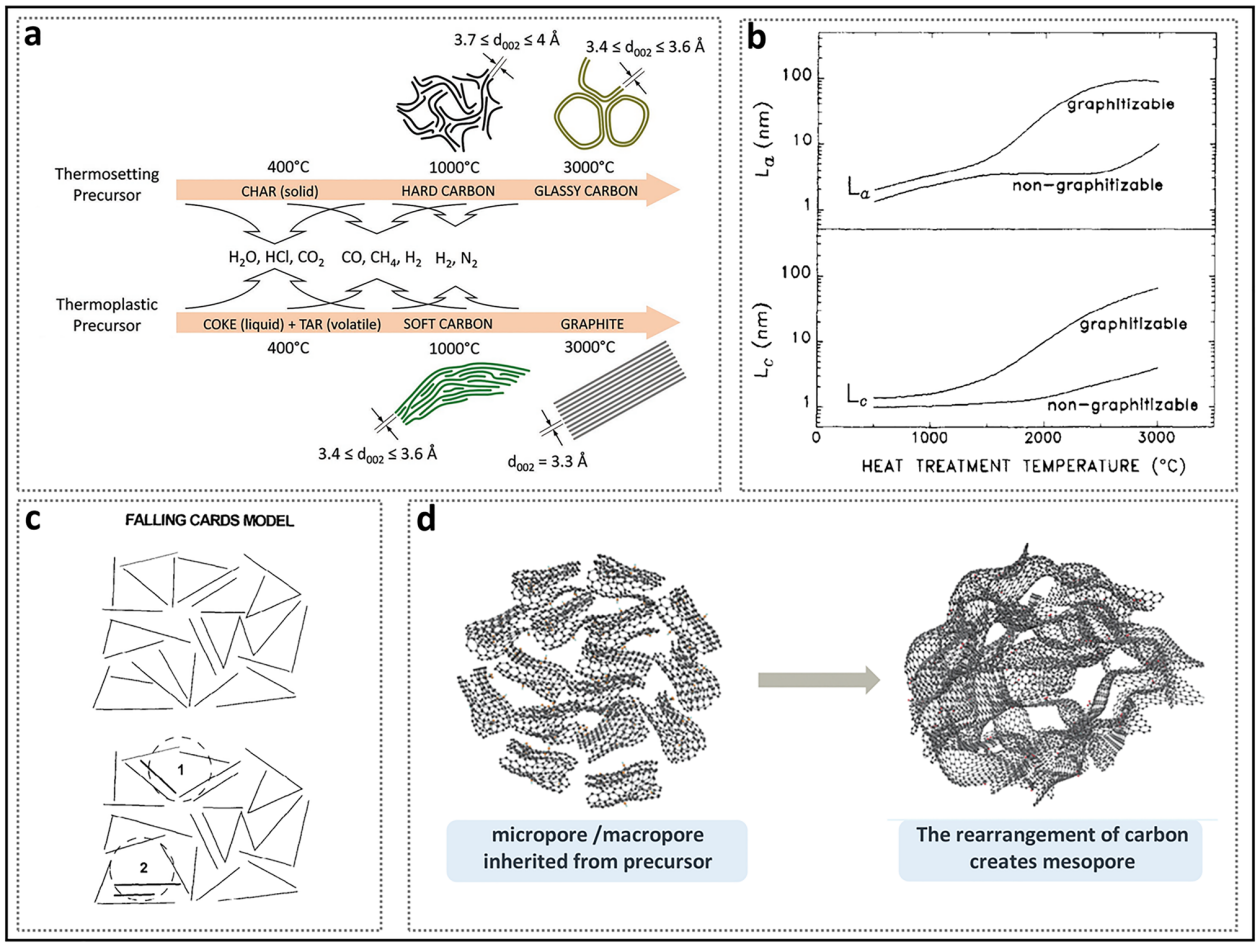
The specific process of carbonization. **a** Schematic representation of the carbonization process during the pyrolysis of thermosetting and thermoplastic organic precursors. Reproduced with permission [77]. Copyright 2018, Wiley. **b** Typical experimental behavior of *L*_a_ and *L*_c_ as a function of heat treatment temperature for graphitizable and non-graphitizable carbons. Reproduced with permission [80]. Copyright 1995, Elsevier. **c** Falling cards model of HC. Reproduced with permission [120]. Copyright 1997, Elsevier. **d** Evolution of pore structure. Reproduced with permission [92]. Copyright 2023, The Royal Society of Chemistry

The formation of open pore structures is originated from three sources: inherent material cross-linking, pyrolytic vapor release, and template removal. Pyrolysis serves as a primary source of open pores in HC, with different precursors displaying different pyrolytic pathways due to significant variations in molecular cross-linking. The heating process supplies sufficient energy to break chemical bonds (such as C=O and O=C–O), resulting in the generation of small molecules and the production of atoms, molecules, or ions with unpaired electrons, i.e., free radicals [118]. Active free radicals can react with other molecules or free radicals, rapidly initiating a series of chain reactions such as dehydrogenation, condensation, hydrogen transfer, and isomerization. As a result of this process, a variety of functional groups are released in the form of volatile compounds (such as H_2_, CH_4_, CO, CO_2_, and NH_3_.), causing a considerable loss of mass (Fig. 13a) [77]. Simultaneously, non-carbon substances are progressively removed from the precursor based on their binding strength, resulting in a continuous increase in the total carbon content. This phenomenon is referred to as the narrow definition of the carbonization process. The vapor is generated from within the material and escapes at the outermost layer, where it in situ forms rigid bulk defects in regions originally occupied by heteroatoms. In general, pyrolysis and carbonization are largely completed before 600 °C and proceed at a significantly slower rate afterward. During subsequent carbonization and graphitization, the cross-linked structure protects the original molecular framework, preventing the complete collapse of the pore structure of HC and preserving a microstructure and morphology similarly to that of the precursor. At higher temperatures, these free radicals still exist and induce the interconnection and rearrangement of six-membered rings to repair the conjugated carbon network, manifested as an increase in the *sp*^2^ carbon ratio [119]. Therefore, it is believed that free radicals dominate the pyrolysis reaction rate and process, playing a crucial role in the conversion of disordered carbon to crystalline forms and in the closure of open pores.

Thermal energy assists in overcoming the graphitization energy barrier, facilitating the transition of carbon phases from an unordered cross-linked structure to graphitic microcrystals. It is worth noting that the formation of crystal nuclei marks the initiation of the graphitization process. The graphitization mechanism includes three basic processes: the growth of microcrystals in the *a*-axis plane, stacking along the *c*-axis, and connection along the a-axis [80]. For better understanding, a comparison of the graphitization processes of soft carbon and HC is presented below (Fig. 13b). Prior to 1000 °C, the crystalline nuclei of both soft carbon and HC progressively increase in size (*L*_a_ and *L*_c_) along both in-plane and perpendicular directions, which results in the formation of numerous small graphene nanosheets with limited size and few-layer stacking. Beyond 1000 °C, soft carbon tends to rapidly stack and interconnect, accompanied by a decrease in the number of microcrystals. This results in the formation of a dense crystalline framework that limits the pore structure from occupying significant space [76]. This intense graphitization reaction is macroscopically manifested as the melting of the intermediate, and thus the precursor is classified as a thermoplastic precursor. In contrast, thermosetting precursors primarily undergo microcrystal growth during the annealing process, exhibiting minimal ordered connection and stacking. The resulting graphite microcrystals consist of numerous few-layer stacks of moderate size, and do not exhibit macroscopic crystalline characteristics. Moreover, the complex, turbulent structure and sustained curvature of HC create optimal conditions for the formation and further development of numerous pores.

The introduction of the “falling cards model” illustrates the specific process through which small-sized pore structures enlarge during graphitization (Fig. 13c) [120]. Driven by sufficient heat, nanosheets shared by two adjacent pore structures can rotate and stack on other nanosheets, forming short-range ordered graphite microcrystalline stripes, which leads to the interconnection of the space occupied by the pores. However, this model overlooks the multifaceted effects of microcrystal size and cross-linking. During the heating process at higher temperatures, defects in the carbon layers are gradually repaired [121]. The size and curvature of the nanosheets continuously evolve, and rotation occurs when spatial constraints are encountered, leading to the coalescence of pores (Fig. 13d) [92]. Moreover, under the combined influence of various defect types, random orientations are maintained, and the growth of dislocated and isolated carbon fragments is delayed, thereby reducing the possibility of excessive crystallinity [40]. It should be noted that very large pore structures are challenging to aggregate and tend to shrink or collapse directly [122].

Upon further heating, the degree of graphitization of the carbon material will continue to increase, making it difficult for the pore structure to aggregate again. This occurs because the nanosheets progressively stack into graphite microcrystals, characterized by reduced interlayer spacing and enhanced interlayer interactions. Graphite microcrystalline ribbons with specific curvatures continue to wrap around the open pores, resulting in larger pore walls and fewer structural voids. When the graphite walls contract to a certain degree, the open pores are predominantly sealed, leaving only minuscule entrances, indicating the transformation of open pores into closed pores. Ideal closed pores of appropriate size can efficiently accommodate a greater number of Na clusters, facilitating the preparation of advanced HC materials with superior overall performance [123]. It is important to note that the sodium-ion accessible diffusion channels are equally crucial, and higher heat treatment temperatures are not always better, as they can lead to the formation of fully closed pores.

#### Possible Issues

As discussed above, the pyrolysis and graphitization processes that form closed pores are closely related to the reactivity of free radicals and the stability of the cross-linked system. Therefore, the carbonization process can be regarded as the result of an integration of chemical kinetics and thermodynamics [124]. Due to their high variability and inherent limitations with respect to different precursor types, several critical issues are often introduced by the traditional direct carbonization method: (i) An insufficient degree of cross-linking that makes the carbon material prone to drastic rearrangement, forming a pore-deficient soft carbon; (ii) open pores with excessively large diameters or overly high concentrations, hindering their transformation into closed pores. These issues can be attributed to the irreversible interplay between thermodynamics and kinetics; (iii) the inappropriate turbulence structure caused by insufficient activation, excessive graphitization, and excessively large pore size hinders the sodium-ion storage efficiency in the closed pores. To enhance the inadequate electrochemical performance of HC, especially their performance in low-voltage plateau capacity, many closed pores structural engineering strategies have been proposed, which will be discussed in detail in the next section.

## Closed Pore Structure Engineering

**Fig. 14:**
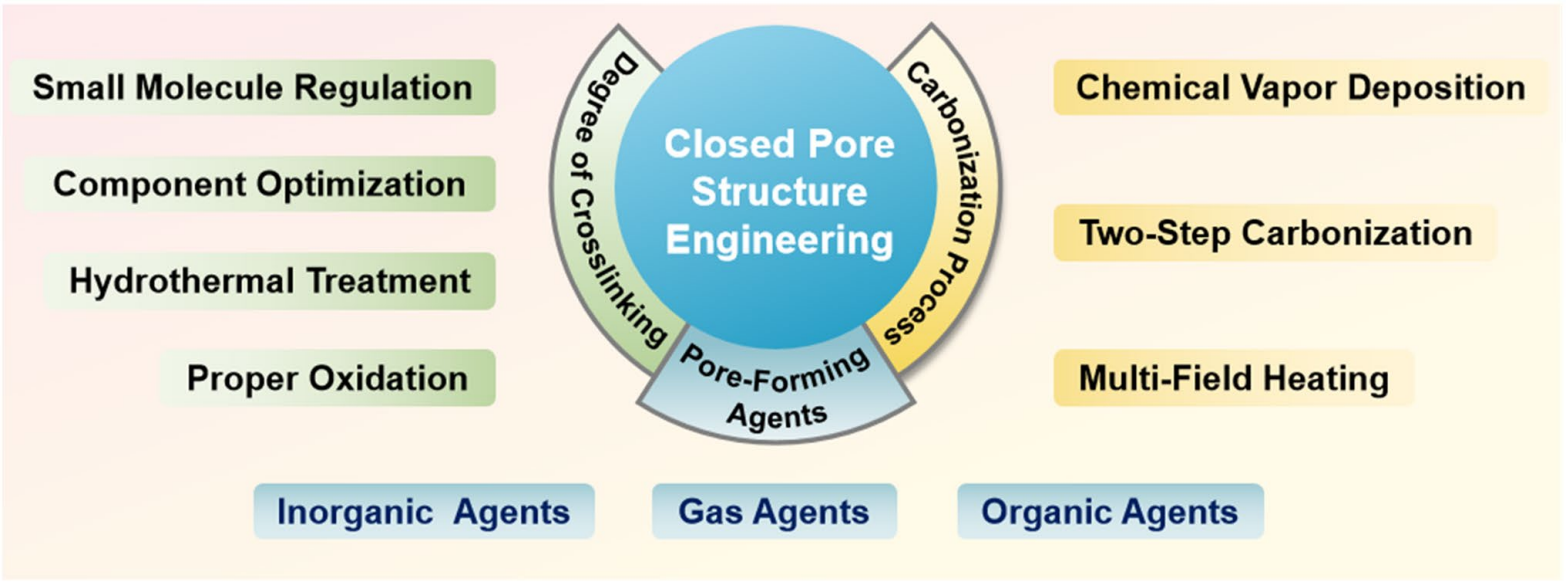
Classification of closed pore structure engineering, including cross-linking degree regulation, pore-forming agent addition, and carbonization process control

Generally, structural engineering of closed pores is categorized into two main approaches based on whether the modification occurs at the precursor stage or during the carbonization process (Fig. 14). Precursor modification involves structural adjustments of the precursor before annealing, such as cross-linking or pore-forming strategies. The cross-linking method enhances the ability of carbon materials to resist graphitization while regulating the turbulence of the microstructure. Meanwhile, pore formation strategies address the issue of insufficient precursor pores. On the other hand, process control refers to regulating the pyrolysis pore formation rate and optimizing the graphitization degree during the carbonization of the precursor, ultimately resulting in the production of high-quality HC. Various factors, including crystal parameter adjustments (*d*_002_, *L*_a_, *L*_c_), molecular structure control, and surface chemistry optimization, are considered to establish a well-regulated system that guides the rational design of closed pore structures (Table 1).

**Table 1:** The impact of various closed pore structure engineerings for different precursors on electrochemical performance

Precursor	Closed pore engineering	Current density (mA g^−1^)	ICE (%)	Plateau capacity (mAh g^−1^)	Total capacity (mAh g^−1^)	References
Bio-oil	Oxidation	20	90.5	~ 220	347.3	[130]
Spores	Oxidation	25	90.23	292.08	453.75	[33]
Phenolic resin	Hydrothermal treatment	20	71.6	~ 280	372.7	[137]
Corn starch	Esterification	30	90.9	~ 240	378	[140]
Phenolic resin	Molecule regulation	30	88.5	241.6	340.3	[142]
Organic molecules	Molecule regulation	50	/	223.7	369.2	[40]
Waste wood	Component optimization	20	/	293	430	[155]
Bamboos	Component optimization	30	92	222	422	[91]
Starch	CO_2_-induced pore formation	25	90.56	351	487.6	[158]
Paper towels	Mn^2+^ reagent pore-forming	20	92.5	226.7	336.8	[60]
Zinc gluconate	Self-template pore-forming	25	91.7	~ 370	464	[34]
Phenolic resin	ZnO template pore-forming	50	77.63	~ 250	501	[172]
Polyacrylonitrile	PEG reagent pore-forming	25	73.7	231.2	365.4	[175]
Ligin	CDs pore-forming	30	78.09	209	368.9	[178]
Phenolic resin	Soft carbon coating	30	74.8	248.2	359.8	[183]
Activated carbon	CVD	25	82.32	490	524	[35]
Phenolic resin	Two-step carbonization	30	83	241.2	351	[191]
Starch	Flash Joule heating	30	/	325	363.2	[46]

### Degree of Cross-Linking Adjustment: Molecular-Level Adjustment of Turbulent Structure

The difference between thermoplastic and thermosetting precursors is frequently attributed to the strength of their cross-linking systems. Cross-linking refers to the process in which multiple linear polymer chains self-assemble and undergo polycondensation, resulting in the formation of a network polymer through the linkage of functional groups on the side chains. In the absence of doped heteroatoms (e.g., N, P), cross-linking in precursors is primarily facilitated via carbonyl and hydroxyl sites. The forms of cross-linking are comprised of non-covalent interactions such as hydrogen bonds and van der Waals forces, as well as covalent bonds including ester and ether linkages.

High-performance thermosetting precursors used in the preparation of HC, which is rich in closed pores are generally plant-based biomass and resins. These oxygen-rich polymers are naturally endowed with a well-developed cross-linking structure from the outset, thereby enabling a controllable graphitization process during sintering and precisely constructing an adjustable pore structure. Plant-based materials not only possess inherent porous structural characteristics but also significant advantages, including abundance, renewability, and environmental friendliness, which are conducive to further industrialization [125]. However, the initial screening process is difficult, and the materials are required to undergo an impurity removal process that is not environmentally friendly before carbonization. Resin-based materials, although expensive and challenging to industrialize, are advantageous due to their consistent raw material properties and straightforward synthesis processes, which render them ideal for molecular design applications.

The three-dimensional cross-linked framework of these precursors inherently contains a limited number of voids, providing additional pore sources for design [126]. During pyrolysis, adjustments to the degree of cross-linking affects both the bond breaking ability of different chemical bonds and the overall rigidity of the skeletal structure. If the degree of cross-linking is insufficient, free radicals are violently released during pyrolysis. This causes excessive pore formation and may lead to the carbon material melting and the loss of its original form [23]. Thermogravimetric analysis (TG) and differential thermogravimetric (DTG) curves indicate that highly cross-linked samples undergo a gentle and uniform pyrolysis process, promoting the formation of low-temperature micropores and high-temperature closed pores [127]. Therefore, the primary goal of pre-cross-linking is to enhance the bonding ability of potential functional groups, thereby regulating the release of vapors and protect the morphology of the generated pore structures. Ultimately, the formation of ideal closed pores is induced.

#### Proper Oxidation

**Fig. 15:**
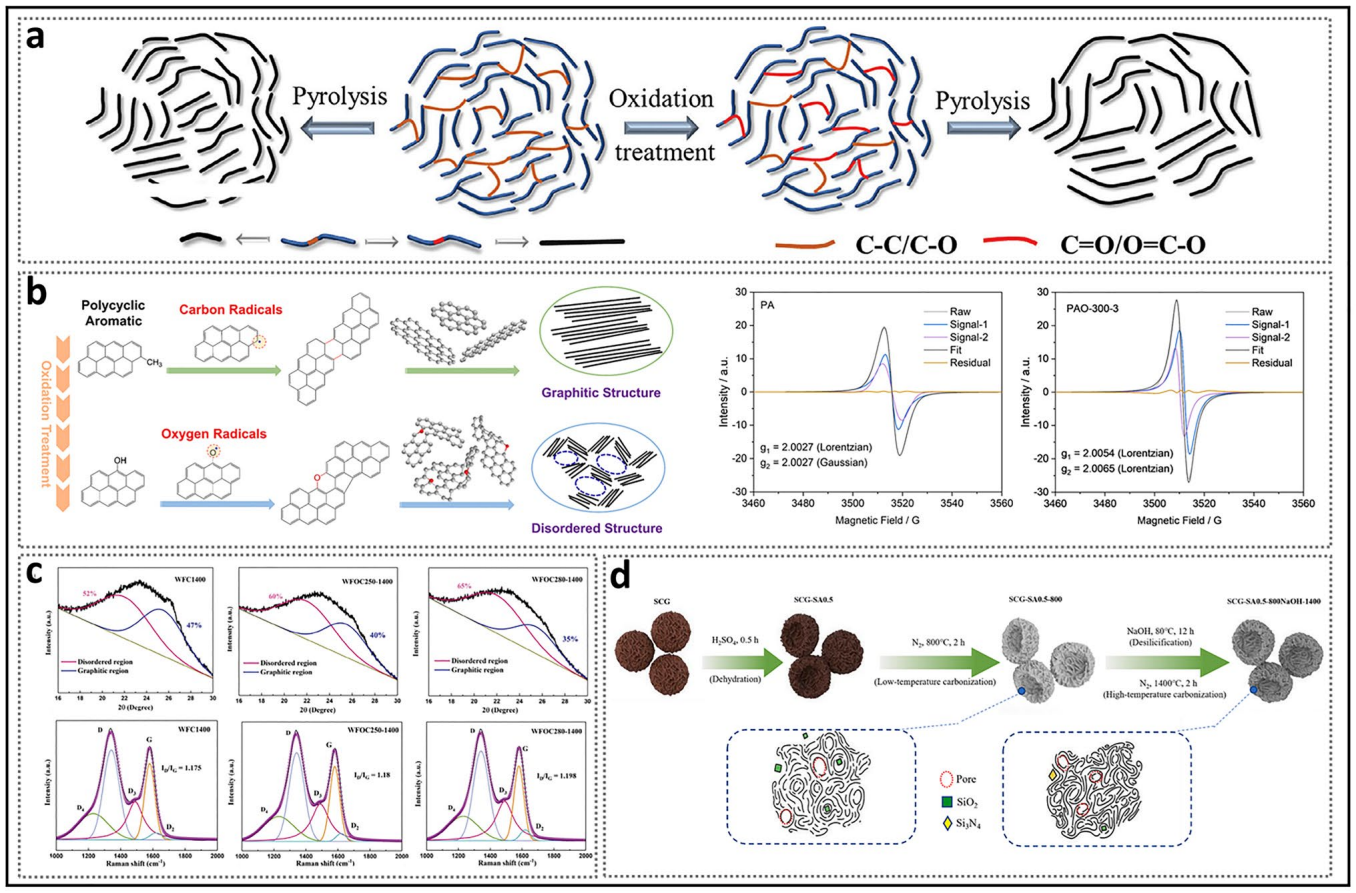
Oxidation strategy modulation. **a** Evolution of functional groups and microstructure during the pyrolysis process. Reproduced with permission [130]. Copyright 2024, Wiley. **b** Mechanism of the HC formation induced by oxygen centered radicals. Reproduced with permission [132]. Copyright 2024, Elsevier. **c** Fitted XRD spectra and fitted Raman spectra of HC with varying oxidation levels. Reproduced with permission [133]. Copyright 2023, Wiley. **d** Preparation process of HC derived from natural spores of Calvatia via a coupling strategy of concentrated H_2_SO_4_ and NaOH. Reproduced with permission [33]. Copyright 2023, Elsevier

Oxidation treatment is a conventional strategy, usually carried out by heating the material in an oxygen atmosphere (< 400 °C) or reacting with oxidizing agents [128]. The oxygen functional groups include carboxyl, anhydride, carbonyl, phenolic, quinone, and lactone groups, each with different thermal stabilities. After oxidation treatment, the abundance and main types of all defects and functional groups can be significantly adjusted, thus influencing the activation level and pyrolysis process of the cross-linked system [129]. In the conjugated network, new oxygen atoms are introduced, and original oxygen functional groups are further oxidized into more stable C–OH or C=O bonds (Fig. 15a), promoting a smoother gas release process and expanding the pore channels in the HC [130]. Due to its high efficiency, oxidation treatment can effectively inhibit the liquefaction of coal tar pitch, which lacks oxygen-containing functional groups, thereby increasing the pathways for the preparation of HC [131]. Wu et al. [132]. studied the effects of the pre-oxidation process on the types of free radicals in petroleum pitch (PA) through electron paramagnetic resonance (EPR) (Fig. 15b). The results showed that some aromatic free radicals, which actively participate in the graphitization process in PA, originated from unoxidized exposed edge sites. After pre-oxidation, the increased g-value of the sample indicated a gradual transition of free radicals toward oxygen-centered types, which compete with the carbon-centered radicals and inhibit their repair into long-range ordered graphite domains. It is worth mentioning that the controllability of oxidation treatment is crucial. Blind oxidation may cause a significant imbalance in the proportions of various functional groups, which in turn hinders the regulation of pore structures. Furthermore, an excess of defects may lead to numerous side reactions. Chou et al. [133] employed a pre-oxidation strategy on the unique waste foam and observed that the disorder of the carbon material increased with the intensity of oxidation (Fig. 15c). Compared to the excellent sample at 250 °C, the sample oxidized at 280 °C exhibited a wider *d*_002_. However, both the ICE and reversible capacity decreased due to the formation of an undesirable SEI. Tian et al. [33] treated spores of Calvatia gigantea precursors with concentrated H_2_SO_4_, causing the collapse of the initial hollow spheres into ideal hollow hemispherical structures (Fig. 15d). After optimizing the carbonization process, the best product exhibited excellent characteristics, such as abundant closed pores and appropriate interlayer spacing, achieving a remarkable specific capacity of 486.0 mAh g^−1^ and an ICE of 90.23%.

#### Hydrothermal Treatment and Esterification

**Fig. 16:**
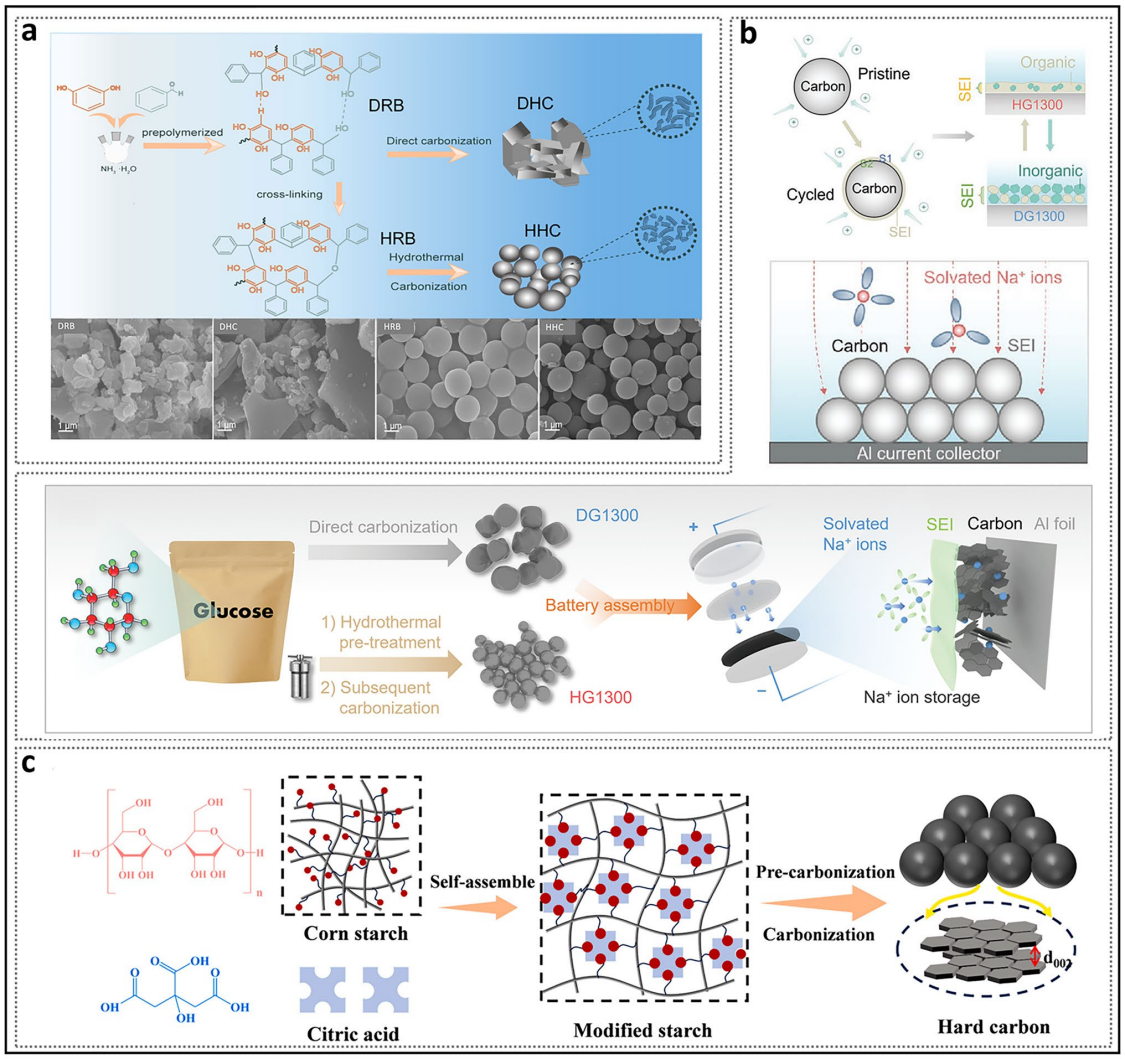
Hydrothermal and esterification strategies. **a** Schematic diagram of the synthetic route and SEM images of DRB, DHC, HRB and HHC. Reproduced with permission [137]. Copyright 2024, American Chemical Society. **b** Effect of hydrothermal pretreatment on SEI and sodium-ion channels. Reproduced with permission [138]. Copyright 2022, Wiley. **c** Diagrammatic representation of the preparation of modified starch-based HC. Reproduced with permission [140]. Copyright 2024, Elsevier

Hydrothermal treatment is a technology with substantial application value in areas such as energy storage, catalysis, and environmental protection. Under low-temperature conditions (< 250 °C), this method facilitates chemical reactions and cross-linking, converting various precursors into monodisperse, low-surface-area, spherical porous morphologies [127, 134]. Compared to oxidation treatments, the hydrothermal method avoids prolonged exposure to air, thereby enhancing beneficial active sites and reducing the formation of unpredictable defects [135, 136]. For example, in the case of phenolic resin, the addition of ammonia water as a catalyst adjusts the polymerization degree of linear phenolic resin molecules, while the solvothermal method plays a crucial role in forming a three-dimensional cross-linked network structure [127]. Song et al. [137] investigated the impact of hydrothermal treatment on the properties of resorcinol–benzaldehyde (RB) resin precursors during the hydrothermal process by controlling the reaction time (Fig. 16a). The results show that with the extension of the reaction time, the cross-linking degree of the RB resin increases, and its morphology gradually becomes spherical. After 6 h, the cross-linking process automatically reaches saturation and stops. Furthermore, Titirici et al. [138] compared two methods: direct carbonization of glucose and hydrothermal pretreatment followed by carbonization (Fig. 16b) They noted that the hydrothermal process appropriately promotes the formation of active sites (such as C=O) in carbon materials. This leads to more uniform decomposition of electrolytes and results in the formation of a more inorganic-rich SEI. At the same time, the spherical morphology and layered porous structure provide shorter and more uniform ion/electron diffusion paths, which improves the kinetics. Moreover, lifecycle assessments demonstrate that this process can enhance the overall carbon yield while reducing carbon emissions, making it a key technology with sustainable potential. Esterification reactions serve as a milder cross-linking method, commonly used to modify starch from preventing the formation of foamed carbon. Due to its natural spherical morphology, starch is an excellent candidate for preparing HC. The unstable glycosidic bonds are prone to breaking, producing many volatile small molecules during pyrolysis, which leads to phenomena such as melting and foaming. This not only destroys the original spherical shape and overall microstructure but also results in low carbon yield and high irreversible capacity. The grafting of ester groups strengthens intermolecular interactions, effectively modifying the thermal chemical evolution mechanism of starch, enabling it to retain a microspherical morphology even after high-temperature treatment [139]. Sun et al. [140] esterified starch with citric acid, which contains multiple carbonyl groups and utilized it as the anode material for SIBs (Fig. 16c). The optimized sample exhibited a closed pore volume of 0.219 cm^3^ g^−1^, delivering a reversible capacity of 378 mAh g^−1^, 90.9% ICE, and outstanding cycling stability.

#### Small Molecule Regulation

**Fig. 17:**
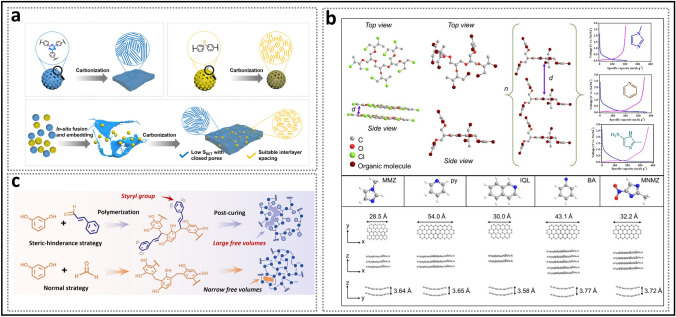
Small molecule cross-linking regulation. **a** Schematic diagram of the synthesis processes of two carbon derivatives with different morphologies and HHC. Reproduced with permission [142]. Copyright 2024, Wiley. **b** Schematic illustration of HC with different spatial steric hindrances derived from small molecule synthesis of the microstructure. Reproduced with permission [143]. Copyright 2024, Wiley. **c** Schematic diagram of the spatial configuration of the polymer, along with the electrochemical properties and crystalline parameters of certain HC materials. Reproduced with permission [40]. Copyright 2024, The Royal Society of Chemistry

Beginning with the polymer source offers a promising research perspective, introducing new factors into the regulation process and helping to reveal the mechanism of closed pore formation in HC. Resin-based precursors are ideal for discussion, as they enable reasonable structural modification of small molecules prior to polymerization [141]. In a study, Wang et al. [142] proposed a design strategy for preparing hybrid hard carbon (HHC) from two carbon materials with different morphologies (Fig. 17a). Due to their relatively weak stability, triazine-cross-linked polystyrene nanospheres will melt in situ into a continuous layered matrix upon heating. In contrast, polystyrene nanospheres with carbonyl groups as molecular bridges can maintain their original morphology. After uniform mixing and co-carbonization, the nanospheres can be embedded into the layered matrix to obtain HHC with a modified framework, characterized by low surface area and abundant closed pores. Xiong et al. [143] first proposed a universal strategy for spatial steric engineering, which involves introducing additional rigid functional groups into small molecules to regulate the cross-linking packing pattern (Fig. 17b). Molecular dynamics simulations confirmed that enhancing steric hindrance increases the free volume in the spatial configuration, facilitating the formation of closed pores during the carbonization process. Additionally, Feng et al. [40] developed an organic synthesis route based on a bottom-up approach to precisely control the molecular spatial configuration (Fig. 17c). When hexachlorobutadiene undergoes nucleophilic substitution, decarboxylation, and polymerization with different nitrogen-containing nucleophiles, the spatial configuration deviates to varying degrees from the original planar configuration, thus affecting multiple crystal parameters of the HC formed during subsequent graphitization. The results indicate that nitrogen-containing side chains and weaker intermolecular forces decrease thermal stability, leading to an increase in the *d*_002_ value of the generated microstructure and the formation of more pores. Additionally, the distortion of the organic molecule’s spatial configuration slightly reduces the *L*_a_ and *L*_c_ values of the finite fragments. Notably, no consistent pattern is observed across multiple samples, suggesting that spatial configuration design is a complex process that must account for several factors, such as heteroatoms, cross-linking systems, and steric hindrance.

#### Component Optimization

With the widespread research on plant-based biomass as precursors for HC, researchers have recognized the crucial role of chemical composition and the component ratio in pore formation [144, 145]. The construction of the primary cross-linking framework of lignocellulose (cellulose, hemicellulose, and lignin) provides a good reversible capacity. The principles of their interaction, however, are still worth further exploration [146]. Additionally, plant biomass typically contains small amounts of other impurities, such as organic compounds (e.g., carbohydrate derivatives) and minerals (e.g., carbonates) [147]. These impurities lead to a significant reduction in electrochemical performance and increase the uncontrollability of the precursor structure, hindering the large-scale application of plant-based biomass. Therefore, optimizing the component structure is an essential approach for modifying plant-based biomass. Depending on the impurities in the precursor, washing and purification are often carried out using deionized water, organic solvents (e.g., acetone), or acid–base reagents (e.g., HCl) [148, 149]. In a similar way, post-washing of the prepared HC is also a feasible measure [150]. Furthermore, researches have shown that rapid heating liquefaction methods (e.g., 100 °C min^−1^) can depolymerize lignocellulose into highly reactive intermediate products, effectively eliminating inherent large pores and undesirable ash content [130, 151].

**Fig. 18:**
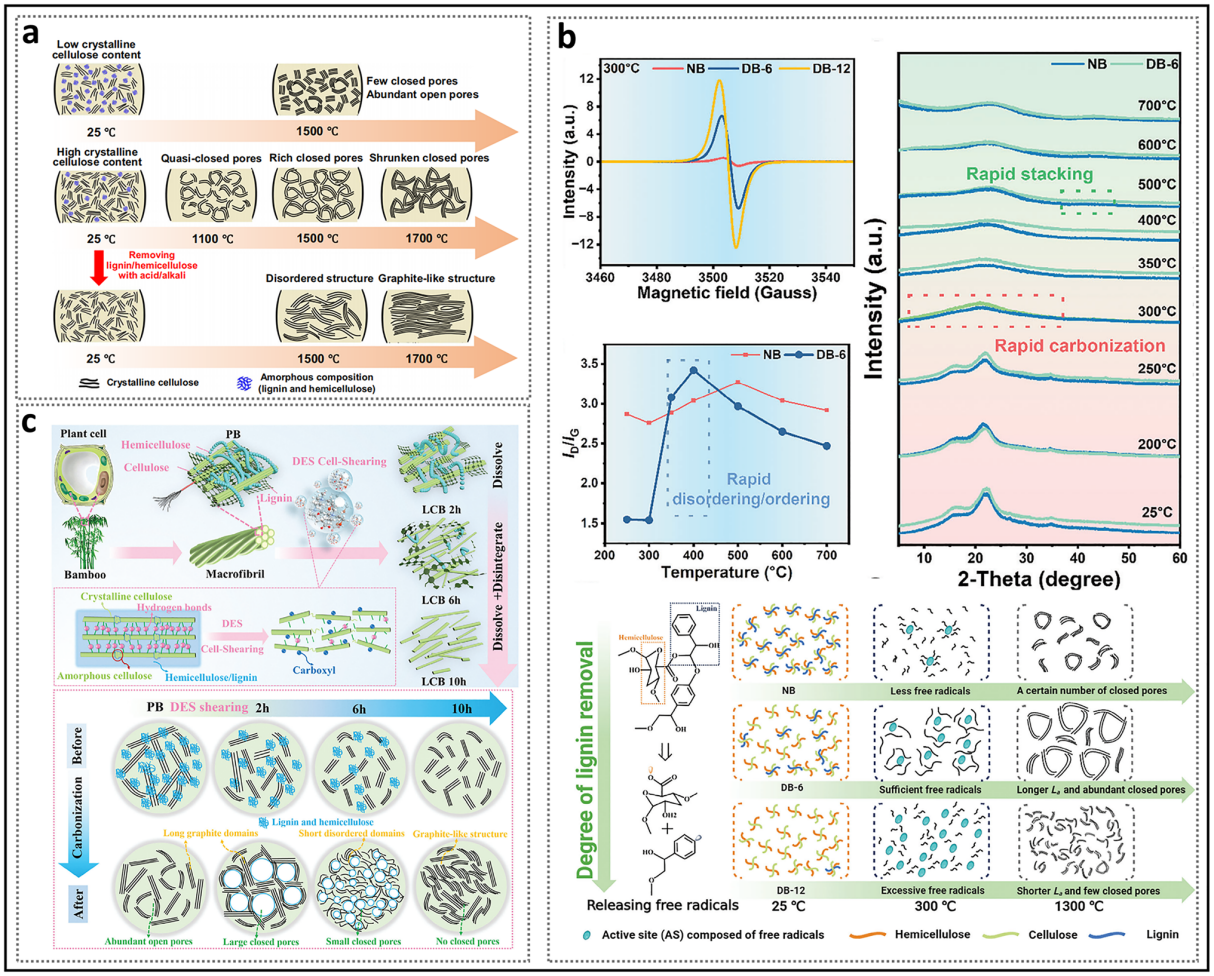
Cross-linking component optimization. **a** Schematic diagram of the formation mechanism of HC closed pores with different compositions of lignocellulose. Reproduced with permission [155]. Copyright 2023, Springer Nature. **b** Influence of free radicals on crystal growth and their guidance in the formation of closed pores. Reproduced with permission [156]. Copyright 2024, Wiley. **c** Schematic diagram of the preparation process of DES cell-sheared cellulose and the effect of different shear times on the microstructure of HC. Reproduced with permission [91]. Copyright 2024, Wiley

Wood and bamboo possess structurally hardened hollow channels, which enable them to serve as potential candidates for self-supporting HC anodes [128, 152]. This phenomenon is attributed to the dense arrangement of crystalline components and the occupation of surrounding amorphous components in the space. However, excessive hardening limits the bending ability of the carbon layer during the subsequent carbonization process, which compromises the precise control of pore parameters [153]. Water washing has little effect on the structure of the precursor, whereas the use of acids or bases alters the three-dimensional structure of lignocellulose, which may either increase or decrease the specific capacity. This has prompted researchers to study the optimization of structural arrangements, and several selective surfactants have been proposed to obtain precursors with different compositional combinations [154]. The important role of various lignocelluloses in the graphitization process has been demonstrated (Fig. 18a): (i) Crystalline cellulose can transform into long-range graphite domains. These domains act as the walls of closed pore structures and can surround active sites; (ii) On the other hand, the presence of amorphous components, such as hemicellulose and lignin, can block the further bonding of surrounding cellulose, thus preventing the formation of over-graphitized HC during high-temperature carbonization [155]. Chen et al. [156] used a mixed solution of NaClO_2_ and CH_3_COOH to selectively remove lignin. This reduced the strength of the cross-linked system while increasing the reaction activity (Fig. 18b). As a result, rapid, disorderly carbonization and early ordered stacking occurred during HC production. EPR analysis showed the content of free radicals released during lignin removal at 300 °C pyrolysis for different samples. These free radicals could accelerate the overall reaction and promote the growth of the carbon layer. In untreated natural bamboo, there were almost no free radicals present, and the final carbon material had smaller microcrystalline sizes. When the free radical concentration was appropriate, it enhanced the tendency of other precursor fragments to combine with them, promoting the formation of a large number of closed pore structures. It is crucial to note that excessive active sites would lead to mutual competition, leading to the formation of dense small-sized microcrystals, which is unfavorable for pore formation. Recently, Zhang et al. [91] introduced a strategy for controlling pore characteristics by shearing bamboo cells with deep eutectic solvents (DES) (Fig. 18c). DES can: (i) dissolve amorphous components (lignin and hemicellulose) to create voids; (ii) remove amorphous cellulose, leading to the breakage of crystalline cellulose chains; (iii) generate competitive hydrogen bonds that weaken the interaction between cellulose molecules, thereby inducing the disintegration of crystalline cellulose chains; and (iv) promote the bonding of carboxylic acids with cellulose, which increases the carbonyl content and enhances the sodium storage kinetics. With an appropriate shearing time, short and monodisperse hollow fibers can form at high temperatures. These fibers have small sizes (≈ 2 nm) and thin pore walls (1-3 layers), achieving a high reversible capacity of 422 mAh g^−1^ and a full-cell energy density of 276.4 Wh kg^−1^. However, excessive shearing completely removes the amorphous components, leaving only the ordered crystalline cellulose, which results in the disappearance of pore structures.

### Pore-Forming Agents for Pore Creation: Efficient Pore Formation

The methods for adjusting the cross-linking degree, as previously mentioned, focus on optimizing the turbulent structure to provide a favorable environment for pore formation. However, the control of crystalline parameters is highly complex and challenging. Furthermore, the sources of pore formation in some precursor materials are still limited. They fail to significantly enhance the plateau capacity. Therefore, the introduction of pore-forming agents (or activators) is widely regarded as the most direct and efficient strategy for enhancing porosity and pore volume in closed pores engineering. It is worth noting that pore-forming agents not only contribute to pore volume, but also enhance the overall degree of disorder, as evidenced by the prominent expansion of the *d*_002_. This further emphasizes the integrity of the microstructure, where various parameters are intricately interconnected.

#### Gas Pore-Forming Agents

**Fig. 19:**
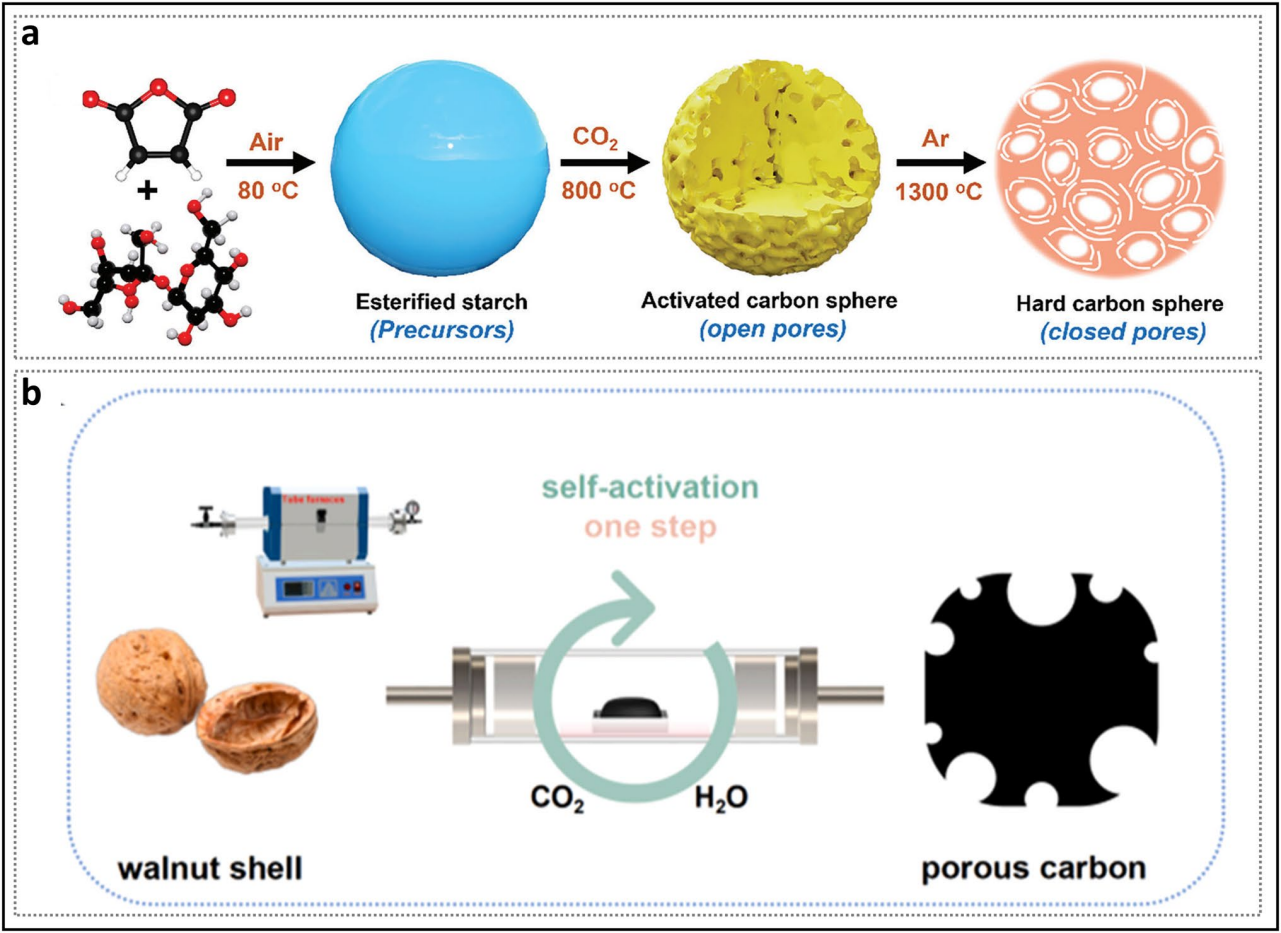
Gas pore-forming agent strategies. **a** Process of CO_2_-assisted pore formation for the synthesis of closed pore HC microspheres. Reproduced with permission [158]. Copyright 2023, Wiley. **b** Schematic diagram of the self-activation process for the preparation of porous carbon. Reproduced with permission [159]. Copyright 2025, Elsevier

Gas activation is a simple process for preparing porous carbon materials. It primarily involves redox reactions between active gases (e.g., H_2_O and CO_2_) and carbon materials at high temperatures. Compared to H_2_O, CO_2_ can react gently with carbon at lower temperatures, making it more suitable for precise pore structure control. For example, Subashchandrabose et al. activated male inflorescences obtained from Borassus flabellifer at 1400 °C using a mixed N_2_ and CO_2_ gas, observing the effects of different gas flow ratios on pore walls and defects [157]. Since activation and graphite repair occur simultaneously in this method, open pores are not fully utilized. Zheng et al. [158]. proposed an improved method for industrial-scale production of HC by separating CO_2_-induced pore formation from high-temperature calcination (Fig. 19a). Specifically, spherical esterified starch precursors with shorter diffusion paths were placed in a CO_2_ atmosphere and reacted at 800 °C to generate CO. After etching, annealing at 1300 °C (in an Ar atmosphere) promoted the rearrangement of carbon layers. This resulted in the preparation of HC with an excellent turbulent structure, exhibiting an extraordinary reversible capacity of 487.6 mAh g^−1^ due to the abundant closed pores with ultramicropores. Additionally, Guo et al. [159] reported a self-activation method in which walnut shells were heated under vacuum (Fig. 19b). This process generated CO_2_, H_2_O, and other small molecules that remained trapped for a long time, effectively producing large surface-area open pores.

#### Inorganic Pore-Forming Agents

In the chemical activation process of porous carbon, a wide variety of inorganic pore-forming agents are used, including acidic substances such as H_3_PO_4_, alkaline substances like KOH, and metal salts like ZnCl_2_. Currently, alkaline substances and metal compounds are the main pore-forming agents used for preparing HC containing many closed pores [160, 161]. Compared to gas pore-forming agents' physical activation, inorganic activators have a stronger etching effect on carbon materials, which can produce rich pore structures.

**Fig. 20:**
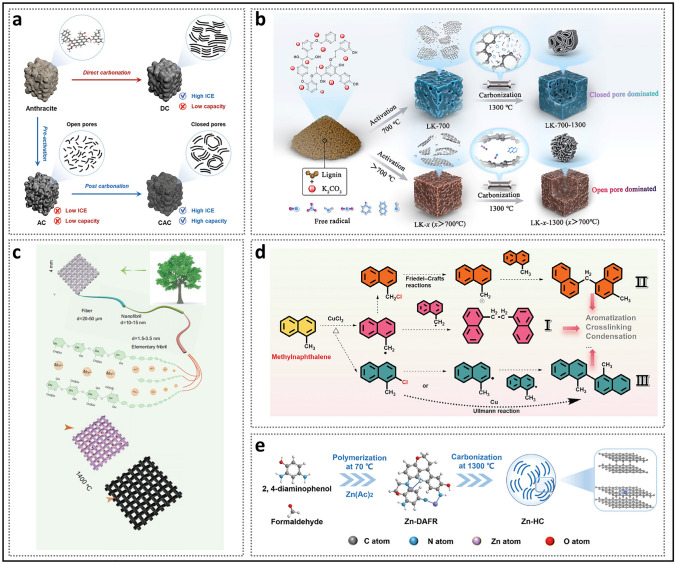
Alkaline substances and metal salt activators. **a** Schematic illustration of the HC synthesis process before and after activation. Reproduced with permission [162]. Copyright 2022, Wiley. **b** Activation and self-healing mechanism in HC. Reproduced with permission [163]. Copyright 2024, Elsevier. **c** Schematic illustration of coordination process between manganese ion and HC precursor. Reproduced with permission [60]. Copyright 2023, Wiley. **d** Schematic illustration of the possible CuCl_2_-mediated reaction mechanism in pitch, using 1-methylnaphthalene as the example. Reproduced with permission [168]. Copyright 2024, Wiley. **e** Schematic illustration of the synthesis of zinc single-atom-regulated HC. Reproduced with permission [169]. Copyright 2023, Wiley

Among alkaline substances, Zhao et al. [162] changed the thermal transformation pathway of anthracite coal by introducing KOH as a pore-forming agent without pre-oxidation (Fig. 20a). This was attributed to the simultaneous inhibition of excessive migration and growth of carbon microcrystals and the creation of microporous structures. Chen et al. [163] used K_2_CO_3_ to activate lignin and studied the effect of different activation temperatures on free radicals during the pyrolysis process (Fig. 20b). The results showed that the content of free radicals gradually decreased with the increase in temperature, leading to carbon skeletons with different stabilities. Based on this, the balance between pore formation and self-repair was effectively adjusted, resulting in the formation of closed pores with expected features.

Additionally, many studies have focused on the pore-forming mechanism of various metals and metal salts [164, 165]. Although metal atoms typically do not directly damage the morphology of carbon, they can influence the formation of the microstructure through various pathways. For example, doping with Fe can catalyze the repair of long-range graphite layers and the formation of nanopores, which is attributed to the formation and subsequent decomposition of Fe_3_C [166]. Moreover, compared to conventional ball milling, the use of metal ions aids in the uniform dispersion of metal elements and better control over pore size distribution. For instance, Goodenough et al. [167] introduced Fe^3+^ through phenolic hydroxyl chelation during the synthesis of phenolic resin-based HC aerogels. This process promoted the transformation of *sp*^3^ carbon to *sp*^2^ carbon and contributed to the development of a porous structure. Moreover, Zhao et al. [60] proposed a metal-regulation strategy using Mn^2+^ to capture oxygen and remove MnO at higher temperatures, aiming to achieve low defect concentration and ideal pore structures (Fig. 20c). However, an excess of Mn^2^⁺ ions not only induce excessive graphitization, which severely disrupts the *sp*^3^ carbon bridges between nanosheets, but also results in the formation of more defects due to migration and escape. Furthermore, certain metal salts can significantly promote the three-dimensional cross-linking of precursor carbon layers. Shao et al. [168] developed an efficient method for converting asphalt into HC (Fig. 20d). The key to this transformation lies in the chlorine atoms coordinated with Cu and Fe, which can significantly adsorb hydrogen and facilitate the chlorination and cross-linking of aromatic compounds. Recently, a method involving the bonding of metal ions with N and other heteroatoms was proposed (Fig. 20e) [169, 170]. This strategy retains certain metal elements in the structure, which has been shown to enhance the sodium storage kinetics and cycling stability of HC.

**Fig. 21:**
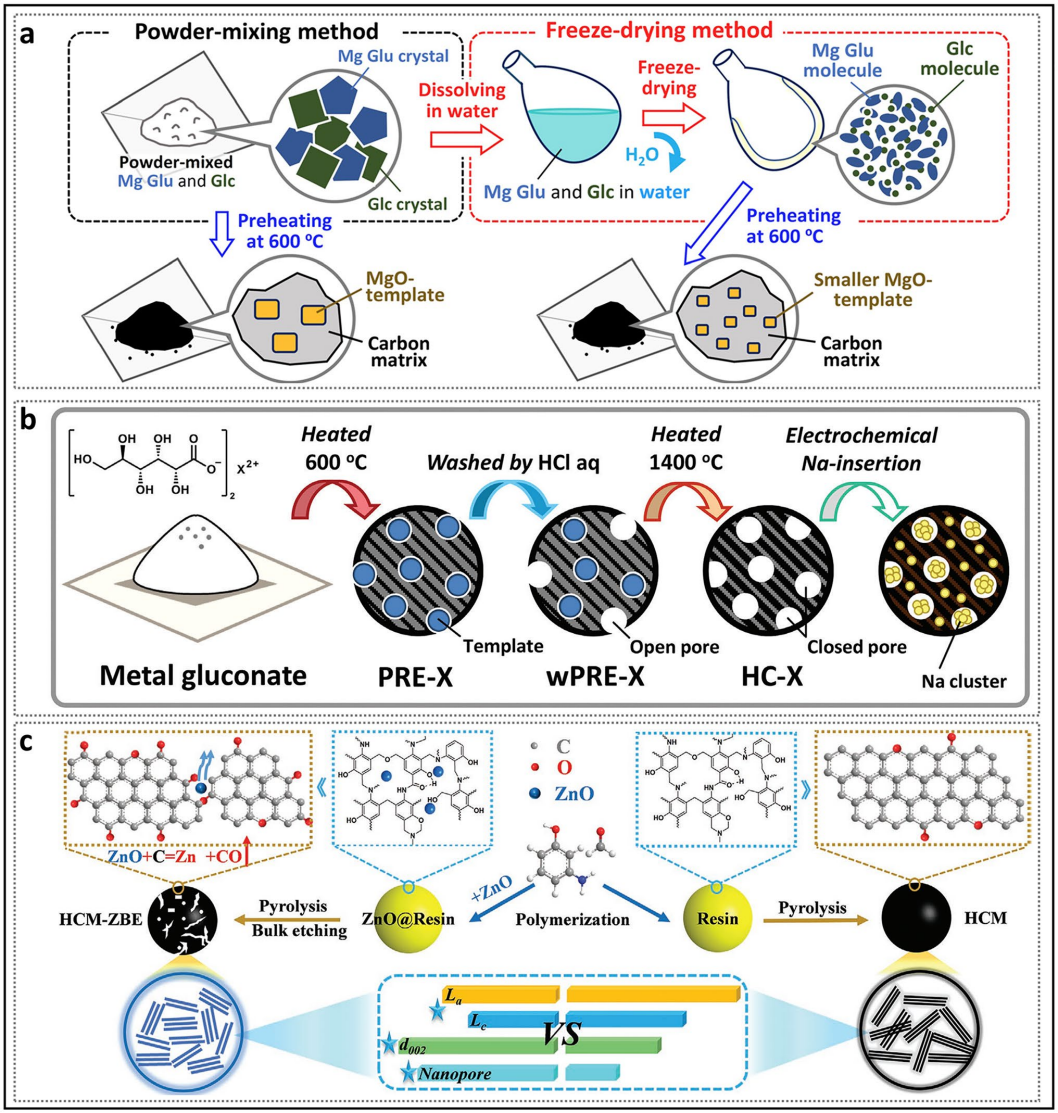
Template-assisted pore formation strategy. **a** Schematic illustration for the two mixing procedures for preparation of the mixtures of Mg Glu and Glc. Reproduced with permission [68]. Copyright 2021, Wiley. **b** Synthesis procedure using metal gluconate as a precursor. Reproduced with permission [34]. Copyright 2023, Wiley. **c** Synthesis route of HC using the ZnO templating method. Reproduced with permission [172]. Copyright 2022, Wiley

Compared to the aforementioned metal salt strategy, the templating method is a superior approach for pore formation. Metal oxides are achieved by directly adding or thermally oxidizing compounds containing metal atoms, a process known as self-templating [59, 171]. During the heating process, the hard template gradually grows to occupy a larger space, which forces the nanosheets to grow around it. Subsequently, an acid treatment is applied to dissolve the metal oxide, allowing for the creation of corresponding pore sizes based on the dimensions of hard template. In a study by Komaba et al. [68], different ratios and mixing processes of glucose (Glc) and magnesium gluconate (Mg Glu) were used to regulate the size and distribution of the MgO template (Fig. 21a). The results showed that a lower activation temperature (400 °C) was insufficient for proper template growth, while 600 °C was the optimal temperature, where the formed MgO was close to its maximum size. Additionally, adding an appropriate proportion of Glc and using freeze-drying can suppress the crystallization of Mg Glu and enhance the dispersion of the MgO template. Furthermore, the team applied this method to zinc gluconate (Zn Glu) and calcium gluconate (Ca Glu) to validate its applicability (Fig. 21b) [34]. They found that due to differences in crystal size, crystallinity, and other factors, the optimal concentration of different metal oxide templates varied. The MgO template HC, made from a 50:50 mix of Mg-Glu and Glc, exhibited a maximum capacity of 478 mAh g^−1^, while the ZnO template HC, made from a 75:25 mixture of Zn Glu and Zn(OAc)_2_, had a maximum capacity of 464 mAh g^−1^. In this case, Glc acts as a template diluent, while Zn(OAc)_2_ serves as an enrichment agent. Additionally, the particle size of CaO or CaCO_3_ generated from Ca Glu reached several tens of nanometers, making it unsuitable as a template for the experiment. Notably, the thermally unstable ZnO can decompose during the formation of closed pores. The CO and Zn vapor produced during the removal process can lead to secondary activation, promoting further expansion of the *d*_002_ plane and the formation of more closed pores. For example, Yin et al. [172] proposed a bulk etching strategy by adding ZnO nanoparticles during the polymerization of phenolic resin, with the optimized sample demonstrating a record reversible capacity of 501 mAh g^−1^ (Fig. 21c).

#### Organic Pore-Forming Agents

**Fig. 22:**
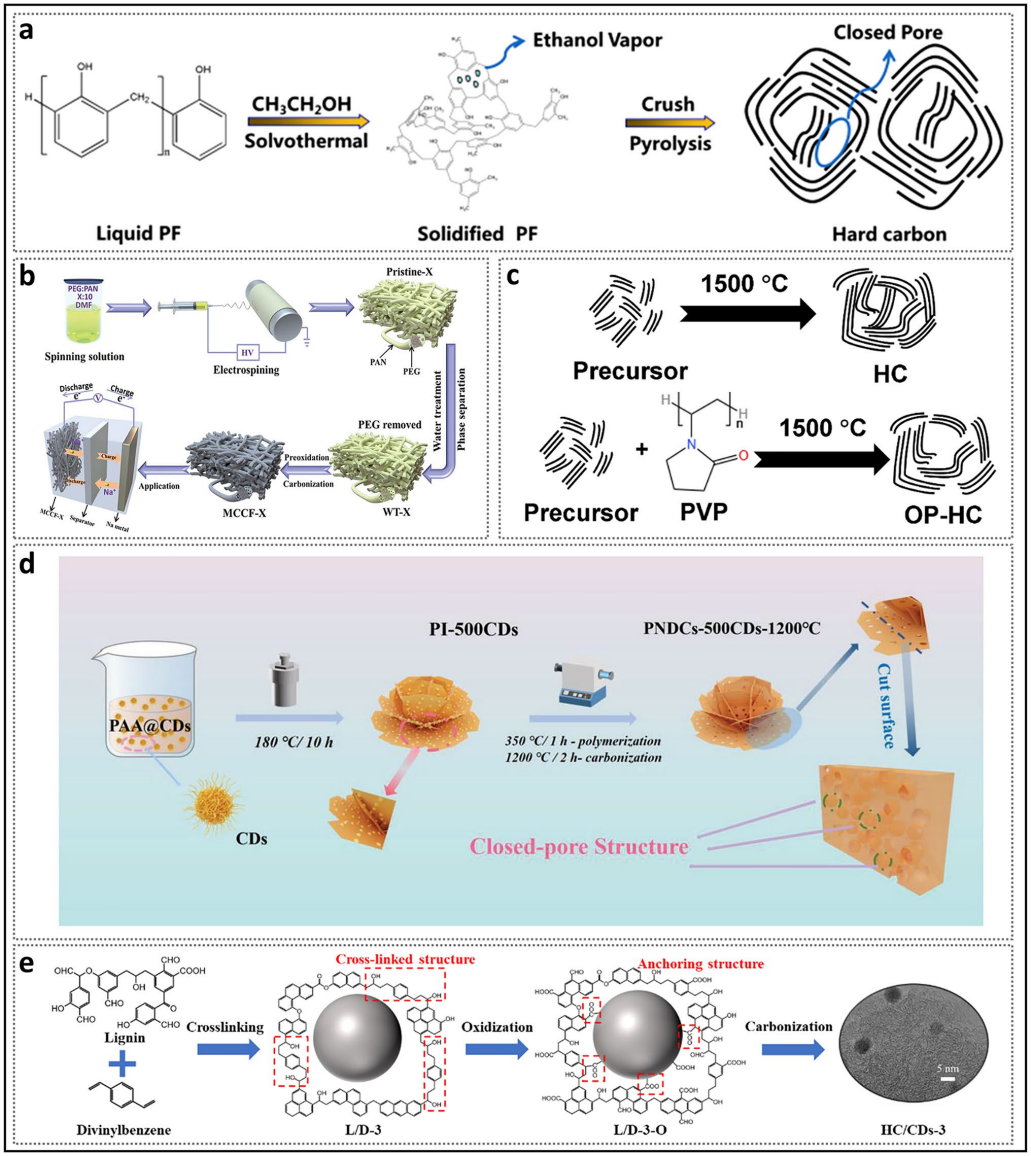
Organic pore-forming agent strategies. **a** Illustration of the typical synthesis process of HC using EtOH as the pore-forming agent. Reproduced with permission [174]. Copyright 2019, American Chemical Society. **b** Schematic illustration of the application routes for samples prepared by mixing PEG and PAN in different ratios. Reproduced with permission [175]. Copyright 2020, The Royal Society of Chemistry. **c** HC with pore channels of different sizes formed before and after the addition of additives. Reproduced with permission [69]. Copyright 2024, The Royal Society of Chemistry. **d** Schematic of the synthesis method using carbon dots as organic pore-forming agents. Reproduced with permission [72]. Copyright 2023, Wiley. **e** Synthesis mechanism diagram of anchoring carbon dots through pre-oxidation. Reproduced with permission [178]. Copyright 2024, Elsevier

Among various methods, the inorganic pore-forming agent strategy is considered to provide the highest reversible capacity. However, the activation process typically requires prolonged washing with water or acid to remove impurities, leading to issues such as high costs and environmental pollution during large-scale production. Given the demands for environmental friendliness and sustainable development, using organic pore-forming agents that do not require washing is a suitable alternative, offering a guideline to enhance the practicality of HC [173]. The creation of pores takes advantage of the poor thermal stability of additives, which decompose and volatilize easily at high temperatures, leaving no residues that could affect performance. For instance, Hu et al. [174] achieved a synergistic balance between phenolic resin curing and ethanol vapor-induced pore opening by precisely adjusting the ethanol content in solvothermal processes (Fig. 22a). The resulting HC, with closed pores after high-temperature treatment, exhibited a large reversible capacity of 413 mAh g^−1^ and an initial Coulombic efficiency of 83%. Additionally, Shi et al. [175] proposed a solvent-induced phase separation (SIPS) method (Fig. 22b), in which the ratio of soluble poly(ethylene glycol) (PEG) to insoluble polyacrylonitrile (PAN) was adjusted to accurately design a multi-channel structure in carbon nanofibers. The presence of this channel structure promoted the expansion of interlayer spacing and the formation of ultramicropores, significantly enhancing ion/electron transport pathways. Meanwhile, polyvinylpyrrolidone (PVP) was also used as an additive to prepare HC derived from waste wood (Fig. 22c), demonstrating the universality of the organic pore-forming agent strategy [69]. As a transition state between organic molecules and inorganic carbon materials, carbon dots (CDs) exhibit extremely high permeability and small sizes [176, 177]. In addition, the surface of CDs is rich in functional groups, and their significant surface effects provide more active sites for sodium storage. Our groupl [72] reported a strategy for in situ introducing kilogram-scale CDs during the hydrothermal conversion of precursors to modulate the closed pore structure (Fig. 22d). When added in small amounts, the CDs primarily undergo pyrolysis on the surface of the carbon material to form ultramicropores. By adjusting the concentration of carbon dots, the porosity and size of the open micropores can be effectively controlled. However, the addition of CDs should be limited to avoid undesirable aggregation phenomena. In addition, Guan et al. [178] introduced a pre-oxidation method, which anchors the CDs onto the HC via oxygen-containing functional groups, successfully preventing self-aggregation at high temperatures (Fig. 22e). It is noteworthy that during the formation process, carbon dots can incorporate other elements (such as nitrogen, sulfur, and fluorine), which not only shows great potential in HC but also offers broad application prospects in other energy storage electrode materials and electrolytes [179–182].

### Carbonization Process Control: Achieving Optimal Structure Through Multilevel Approaches

After the precursor has been successfully modified to achieve enhanced cross-linking characteristics and porosity at the microscopic level, the next crucial step involves the high-temperature calcination process in an inert atmosphere, with the aim of introducing more rational open pores and further evolve closed pores. Factors such as heat treatment temperature, heating rate, and duration are known to significantly affect the final structure and texture of the HC. Among these, the final annealing temperature is the commonly explored starting point. Precursor materials containing a high concentration of open pores (e.g., porous carbon) are typically required to undergo very high calcination temperatures, making them unsuitable for practical applications. Furthermore, if the heating rate and duration are not modified, the carbonization process is primarily governed by the thermodynamics of the graphitization reaction. A lower temperature might result in incomplete closure of some pore openings, while a higher temperature presents the risk of excessive defect healing and the formation of closed pores, significantly limiting the controllability of the microscopic structure. Therefore, to fully exploit the potential of hard carbon, it is essential to adopt a combination of various carbonization techniques to achieve a comprehensive optimization of both its structural properties and electrochemical performance.

#### Chemical Vapor Deposition: Space Confined Customization of Closed Pores

**Fig. 23:**
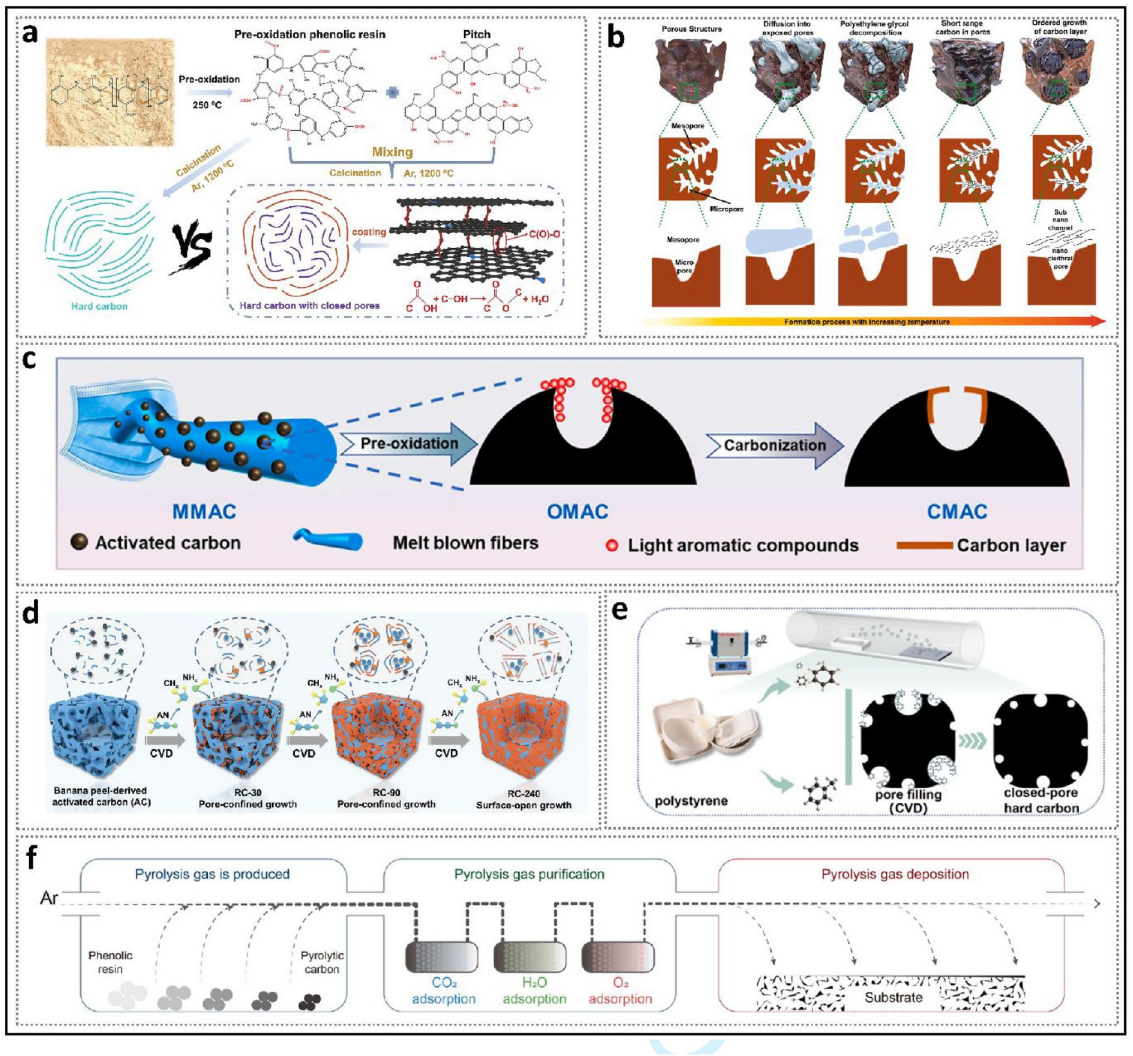
Spatial confinement strategy. **a** Schematic illustration of the soft carbon coating strategy. Reproduced with permission [183]. Copyright 2024, Wiley. **b** Mechanism of confined carbonization with formation of nano cleithral pore and sub nano channel. Reproduced with permission [58]. Copyright 2024, Elsevier. **c** Schematic diagram of using waste mask activated carbon to reduce the closed pore entrances. Reproduced with permission [184]. Copyright 2024, Elsevier. **d** Schematic diagram illustrating the effect of different deposition times on pore structure. Reproduced with permission [35]. Copyright 2024, Wiley. **e** CVD process through polymer pyrolysis gasification under vacuum conditions. Reproduced with permission [159]. Copyright 2025, Elsevier. **f** Schematic diagram of high-value utilization of pyrolysis gas. Reproduced with permission [186]. Copyright 2024, Elsevier

A feasible approach to regulate porous carbon with an ultra-high specific surface area is to increase the content of short-range ordered graphite domains through in situ deposition, utilizing the space confinement effect to promote the formation of closed pore necks. In situ deposition refers to the process of mixing heat-sensitive materials (such as soft carbon (Fig. 23a) or organic polymers (Fig. 23c)) with a porous carbon substrate during heating [183, 184]. These materials melt and enter the porous carbon as the temperature rises and then undergo pyrolysis and deposition to form graphite carbon (*d*_002 _≈ 0.36 nm). The deposition effect can be controlled by varying the amount of the additive. However, this melting modification strategy lacks precision due to the uncontrollable nature of the pyrolysis process of the additive. Wu et al. [58] pointed out that when reagents with larger molecular weights are used, the pyrolyzed carbon does not directly enter the pores but rather deposits at the pore channels (Fig. 23b). In other words, pyrolyzed carbon inevitably deposits on the surface, which not only reduces the specific capacity but also lowers the deposition efficiency. Therefore, high-temperature assistance (> 1200 °C) is essential, though it may cause some micropores to transform into mesopores. These issues highlight the relatively low potential upper limit of this strategy.

Building on this approach, chemical vapor deposition (CVD) has been proposed for structure engineering of closed pores. This method involves introducing small molecular gas flows (e.g., methane, benzene, toluene) at energy-efficient temperatures (usually 700-900 °C) to complete the deposition on a substrate, modifying the nanopore characteristics [185]. By controlling the CVD duration, efficient customization of closed pores can be achieved and applied to porous carbon precursors with any activation levels. The improved carbonization process offers the following advantages: (i) gases can be adsorbed through van der Waals forces near ideal pore active sites before undergoing pyrolysis, reducing adverse deposition on surfaces and morphological changes; (ii) mild working conditions prevent excessive growth of graphite domains, maximizing the space confinement effect with minimal impact on the overall turbulent structure; (iii) ultra-micropores and micropores are more likely to be preserved, without significant aggregation. Based on this, Fan et al. [35] prepared an anode material with a smooth surface, achieving a reversible capacity of up to 524 mAh g^−1^ (plateau capacity of 490 mAh g^−1^) (Fig. 23d). In addition, Guo et al. [159] simultaneously heated unblended porous carbon and polystyrene in a vacuum atmosphere and achieved a similar CVD effect using pyrolyzed gases from polystyrene (Fig. 23e). Zhang et al. [186] collected the mixed gases from resin pyrolysis, purified them and then used them for CVD **(**Fig. 23f). These works demonstrate the excellent scalability and economic feasibility of the CVD process.

**Fig. 24:**
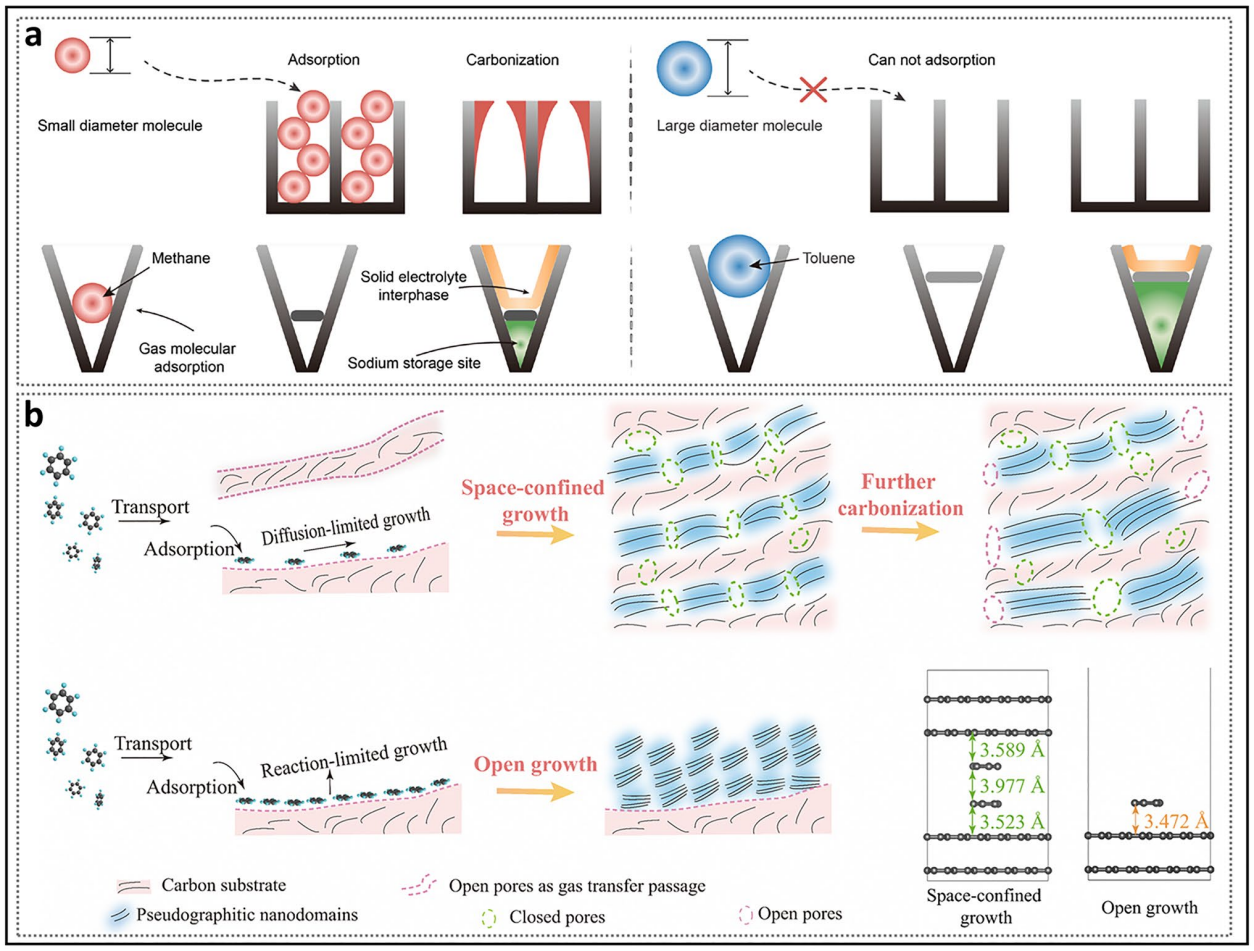
The mechanism of closed pore formation via CVD preparation. **a** Selectivity of pores for gas molecules during the CVD process. Reproduced with permission [186]. Copyright 2024, Elsevier. **b** Two growth mechanisms of benzene pyrolyzed carbon and the corresponding calculations of the interlayer spacing between deposited carbon layers and carbon substrates. Reproduced with permission [187]. Copyright 2023, The Royal Society of Chemistry

A key discussion point in this approach is the mechanism by which deposited carbon induces the transformation of open pores into closed ones. Based on some experimental phenomena, two different viewpoints have been proposed. One perspective, building on the concept of sieving carbon introduced by Yang et al. [61] suggests that the deposited carbon mainly acts on the surrounding entrances of the pore walls. As pyrolysis progresses, the pore defects continuously shrink until gas-phase molecules can no longer pass through, at which point the reaction spontaneously terminates. Therefore, it can be inferred that the pore diameter at the gap of the prepared closed pores is approximately equal to the kinetic diameter of the chemical molecules used. In the experiment conducted by Zhang et al. [186], by comparing the electrochemical performance of CVD with different carbon sources, it was highlighted that the type of carbon source plays a crucial role in determining the closure of pore entrances (Fig. 24a). The larger molecular diameter of toluene provided superior reversible capacity and ICE because it was hindered from entering the micropores of the HC, leaving more effective space for sodium storage. In contrast, methane with a smaller molecular diameter easily penetrated deeper into small-sized pores, leading to excessive closure of the pore structure and a reduction in the volume and proportion of micropores. While this is easy to understand, sieving carbon is considered for each pore individually, without an explanation of the pore channels, so it does not provide a clear insight of the microscopic structure. Cao et al. proposed another concept of filling carbon, suggesting that the inner pore walls are the key sites for catalytic adsorption reagent decomposition, leading to the formation of wrinkled graphite-like fragments in slit-shaped pores (Fig. 24b) [187]. Defect-free graphite carbon tends to stacks more layers, restricting the transport path for electrolytes, thereby splitting open pores into multiple closed ones. It is important to note that as the deposited carbon continues to grow, the pore channel space gradually compresses, leading to a sacrifice of total pore volume. If the open pores are fully converted, further pyrolysis of the carbon may lead to excessive graphitization, which will begin to reduce the sodium storage performance. Notably, this study found that post-heating after CVD (at 1300 °C) significantly improved the reversible capacity (from 348.0 to 435.5 mAh g^−1^), attributed to the expansion of closed pores. This indicates that CVD customization for closed pores is not perfect and still has significant room for improvement. Furthermore, to verify the principle of space confinement, this work compared with mesoporous carbon. Experimental and DFT calculation results show that the growth of pyrolytic graphite in mesoporous carbon is governed by surface reactions. In larger spaces, the pyrolytic carbon arranges in small, stacked layers, leading to the complete blockage of the mesopores. Based on this, it can be concluded that the pore size and uniformity in different materials have a significant impact on the final CVD effect. Micropores, when dominant, can be better repaired into closed pores.

#### Two-Step Carbonization and Multi-Field Heating

**Fig. 25:**
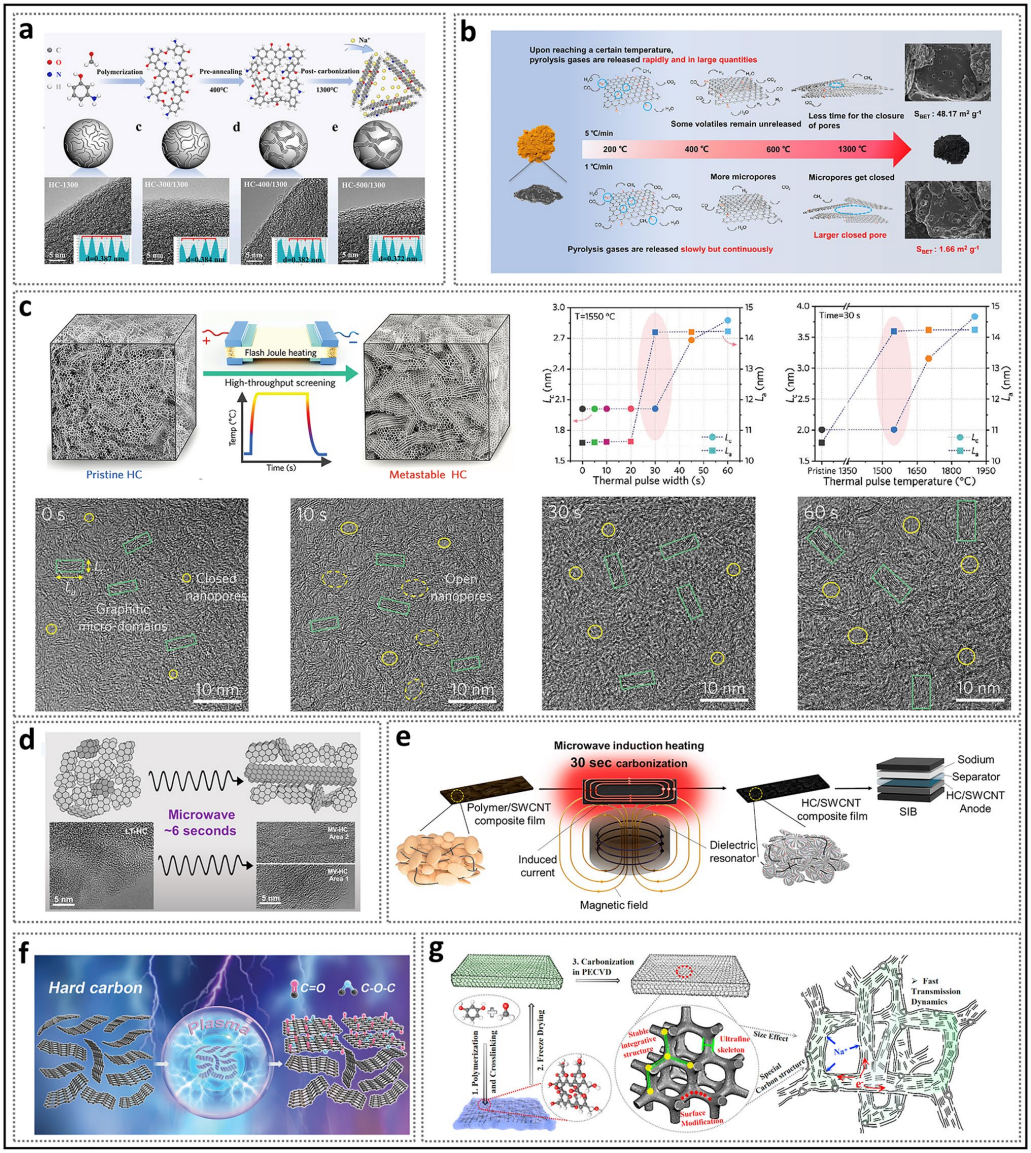
Various modulations of the carbonization process. **a** Flowchart of the two-step carbonization process for preparing HC, along with HRTEM images at different pre-carbonization temperatures. Reproduced with permission [191]. Copyright 2024, Elsevier. **b** Schematic illustration of the structural evolution of carbon materials at different heating rates. Reproduced with permission [196]. Copyright 2024, The Royal Society of Chemistry. **c** The impact of flash Joule heating strategy parameters on the microstructure. Reproduced with permission [46]. Copyright 2024, Wiley. **d** Reproduced with permission [201]. Copyright 2018, American Chemical Society. **e** Schematic of the ultrafast preparation of HC anodes via microwave induction heating of polymer/SWCNT composite films. Reproduced with permission [202]. Copyright 2024, Elsevier. **f** Schematic illustration of the low-temperature oxygen plasma treatment. Reproduced with permission [208]. Copyright 2022, The Royal Society of Chemistry. **g** Schematic illustration of the integrative carbon network preparation process. Reproduced with permission [209]. Copyright 2022, Elsevier

As the free radical theory gains more attention, controlling the evolution of the microstructure of HC from a kinetic perspective has become an effective and direct research approach. In simple terms, refining the details of pyrolysis or graphitization processes can adjust the parameters of crystals and closed pores, leading to superior electrochemical performance [188]. Based on this concept, a two-step carbonization strategy, which includes an additional low-temperature annealing process, has been proposed with the goal of independently separating the pyrolysis and graphitization stages (with some overlap still present) [189, 190]. During the first carbonization step (also referred to as the pre-carbonization step), the overall framework stabilizes after pyrolysis. Meanwhile, the degree of defect repair and the ordering of graphite microcrystals increase, attributed to the ample energy and time provided for the free radicals to react (Fig. 25a) [191]. The set temperature (< 800 °C) is selected based on the expected pyrolysis rate and material thermal stability, enabling precise control of the thermal process [192, 193]. Following this, a subsequent high-temperature calcination (> 1000 °C) is carried out to graphitize the material and obtain the final form and structure of carbon materials with improved morphology and structure. Pre-carbonization is highly operable and adjustable, making it suitable for various HC preparation processes, especially for balancing the defects introduced by pre-oxidation treatments [88, 194, 195]. Meanwhile, the heating rate plays a crucial role in controlling the intensity of pyrolysis, as a high rate can cause pyrolysis gases to be produced quickly without being effectively released. Tang et al. [196] employed a slow pre-carbonization strategy for gentle pyrolysis, resulting in carbon intermediates with larger specific surface areas and abundant, uniform micropores (Fig. 25b). At the same time, from another perspective, increasing the pyrolysis duration leads to a greater tendency for these micropores to close. Similarly, gas flow rate affects the residence time of pyrolysis gases, thus influencing the secondary activation of small molecules like CO_2_ and H_2_O [20].

Although the two-step carbonization method is ideal for regulation, the additional steps and slower pyrolysis rate introduce longer reaction times, significantly extending the experimental period and increasing energy consumption. In contrast, multi-field methods such as flash Joule heating, microwave irradiation, and spark plasma sintering (SPS) can achieve rapid temperature rise, allowing the synthesis of carbon materials (such as graphene, carbon nanotubes, and HC) under fast, energy-efficient, and sustainable conditions [197]. Flash Joule heating is a process where a pulsed current is passed through a sample, generating a large amount of heat instantaneously via the Joule effect [198]. Paired with a cooling system, this process can switch between room temperature and over 1000 °C in just a few seconds [199]. Compared to traditional slow heating, the synthesized HC has smaller particle sizes, larger interlayer spacings, and higher closed pore concentrations [199]. Moreover, Zhang et al. [46] applied this strategy to post-processing commercial HC. By adjusting the pulse temperature and width, they systematically studied the evolution of the carbon phase under kinetic conditions (Fig. 25c). At a fixed temperature of 1550 °C, the *L*_a_ increased sharply between 20 and 30 s, while the *L*_c_ began to grow rapidly after 30 s. The sample HC (30 s) effectively avoided severe stacking of carbon layers, resulting in in-plane expanded short-range ordered graphite microdomains. Regarding closed pores, HRTEM showed that at HC (10 s), pore size increased, forming some open nanopores, which completely closed at 30 s, resulting in the highest plateau capacity. Beyond this point, the overdeveloped pore walls inhibit the potential for sodium storage in closed pores, causing the specific capacity to decrease rapidly. Similarly, under a fixed heating time, the temperature at which *L*_a_ changed significantly was lower than *L*_c_, indicating the feasibility of directional regulation of crystallite characteristics. Furthermore, the strategy of using microwave radiation to induce molecular vibrations inside the material has also been studied [200]. For example, Ji et al. [201] achieved a significant improvement in the reversible capacity of HC (from 204 to 308 mAh g^−1^) and superior electronic and ionic conductivity compared to conventional carbonization, by simply applying a 6-s microwave treatment after pre-oxidation and pre-carbonization of the filter paper (Fig. 25d). Subsequently, Park et al. [202] designed a controllable microwave device that contributed to the scale-up production of HC anodes (Fig. 25e). Meanwhile, microwaves can also assist in rapid hydrothermal processes, leading to the formation of layered porous structures, including ultramicropores [203, 204]. Inspired by flash Joule heating and microwave heating, the SPS process assisted by high-energy plasma, hot pressing, and resistive heating was proposed [205, 206]. Various carbon sources can directly be converted into HC at a heating rate of 300 °C min^−1^ [207]. However, due to the overly intense pyrolysis reaction, the oxygen content of the sample decreases significantly compared to conventional carbonization, accompanied by a substantial reduction in the *d*_002_ value. Shen et al. [208] used low-temperature oxygen plasma technology to customize the C=O functional groups on the HC surface, increasing the sodium storage active sites without significantly changing the *d*_002_ (Fig. 25f). Specifically, Wu et al. [209] employed plasma-enhanced chemical vapor deposition (PECVD) to adjust the stacking structure of nanofibers in phenolic resin aerogels, obtaining a new type of integrated carbon network material (Fig. 25g).

## Summary and Prospect

In recent years, the discovery of closed pore structures has greatly enhanced the research vitality and application potential of HC materials. Both opportunities and challenges are present, with substantial research focused on addressing key issues, such as graphitization degree, crystal parameters, disordered stacking structures, and closed pore features. This review summarizes the latest research progress in this field, first providing a detailed explanation of the crucial role of closed pore structures from a mechanistic standpoint. Through an in-depth analysis of sodium storage mechanisms, the key characteristics of closed pore are systematically revealed. Secondly, various design strategies for closed pore structures are reviewed, providing valuable guidance for future structural regulation. Despite the substantial progress, the current understanding of HC remains limited, and many theoretical perspectives still lack consensus, placing higher demands for future research. To promote the long-term development of SIBs energy storage applications, several new and effective viewpoints are proposed.

### Unified View of Active Sites

In addition to the classical two-stage models, the introduction of three-stage and hybrid models has expanded the possibilities for exploring global mechanisms pathways. This raises an important question: why do HC exhibit such diverse properties? Is it necessary to offer multiple explanations to fully elucidate their characteristics? Previous studies primarily focused on independent analyses of defects, carbon layers and pores, using theoretical calculations to explain various sodium storage mechanisms. Although current in situ and ex situ techniques have provided a wealth of data, contradictions between these methods have hindered the clarification of essential differences among various HC. This suggests that a mindset based on fixed structures might have certain limitations. In this review, we have mentioned a relationship between three active sites: (i) Larger interlayer spacing promotes pseudocapacitive adsorption, suggesting a close correlation between nanosheet size and the presence of heteroatoms and edge defects; (ii) During the filling of closed pores, adsorption on the pore walls serves as a prerequisite, followed by the formation of quasi-metallic sodium clusters in accordance with the thermodynamic trends of pore sizes; (iii) closed pores can be viewed as carbon nanosheets that expand in the middle, and the filling mechanism occasionally exhibits diffusion behavior similar to intercalation. Therefore, it can be concluded that a synergistic effect and overlap exist between these three active sites. As the most clearly defined structure, closed pores are anticipated to become the core of future theoretical research, advancing the organic unification of the three mechanisms.

### Structure–Performance Correlation from a Molecular Perspective

Due to the amorphous characteristics of HC anodes, their reproducibility is limited, and establishing a clear correlation between structure and performance remains challenging. Therefore, comparative studies using consistent, systematic raw materials are crucial. Research on resin precursor and the optimization of biomass precursor components have highlighted that exploring the properties of HC from a molecular perspective is both feasible and essential. However, the quantification of specific parameters remains a significant challenge. Many key characterizations depend on average values, which fail to accurately represent the overall distribution of material properties. Furthermore, the characterization framework for closed pore features is still underdeveloped, particularly in situations where gas adsorption methods are inapplicable, underscoring the urgent need for more precise and comprehensive characterization techniques.

### In-Depth Study of Pore Structures

Understanding how open pores transform into corresponding closed pores is a key aspect of studying these pores. Pyrolysis and activation processes play a pivotal role in the formation of active carbon and the engineering of closed pores. While numerous experiments have used commercial activated carbon as a precursor and obtained certain results, this approach restricts further exploration of the full potential of closed pores. Factors such as activation efficiency, pore morphology, and structural models are directly related to the mechanisms underlying the closure of pores, thus constituting a significant area for in-depth investigation. Currently, much of the knowledge about closed pores is derived from HRTEM, which can partially reveal possible structures of closed pores. However, two-dimensional characterization methods impose limitations on the direct visualization of topological structures. Although the precise morphology of closed pores cannot be directly observed, existing studies have shown the presence of pore gaps and channels. The classification scheme proposed in this review—"quasi-closed pores," "closed pores," and "fully closed pores"—is expected to facilitate a more comprehensive analysis of the morphology and formation processes of closed pores. Moreover, the influence of closed pore features on rate performance remains to be thoroughly investigated, and future studies may systematically integrate electrolyte engineering.

### Design Directions for Closed Pore Structure Engineering

Numerous studies have demonstrated that achieving high reversible capacity is strongly linked to the closed pore structure. However, several factors influence the performance of closed pores, and understanding how to effectively optimize the design across various aspects is critical. Therefore, this review provides an overview of the ideal directions for closed pore design:

i. Molecular-level optimization of precursor configuration: Firstly, design molecular structures and compositions to modulate steric hindrance and stacking behavior. Secondly, adjust the cross-linking degree to achieve optimal thermal stability and particle morphology.
ii. Introduction of suitable pore formers: On one hand, explore ideal pore sizes to accommodate sodium clusters and create open pores with high concentration. On the other hand, develop eco-friendly, efficient pore-forming agents to ensure high-quality, sustainable development.
iii. Integration of kinetics and thermodynamics: Precisely control phase transitions and final turbostratic structures to create appropriate pore entrances (providing plateau capacity), regulate uniform pore size distribution (enhancing filling efficiency), and establish effective diffusion pathways (avoiding fully closed pores).
iv. Defect engineering: Firstly, closely integrate the pore entrances and control the concentration, type, and reversibility of defects to promote the filling mechanism of closed pores. Secondly, investigate the precise regulation of heteroatom functional groups to form favorable SEI.
v. Electrolyte engineering: Explore the influence of the electrolyte from both the SEI and solvation processes to further enhance the understanding of the definition and structure of closed pores. Design a well-optimized electrolyte to induce the optimal solvation structure, while forming a thin and stable SEI to improve rate performance and cycling stability.

In conclusion, due to its robust low-potential charge–discharge plateau and excellent desolvation ability, HC with advanced closed pores exhibit great potential in SIBs and are expected to become the most competitive high-performance anode materials in future.
